# Predicted impact of thermal power generation emission control measures in the Beijing-Tianjin-Hebei region on air pollution over Beijing, China

**DOI:** 10.1038/s41598-018-19481-0

**Published:** 2018-01-17

**Authors:** Liqiang Wang, Pengfei Li, Shaocai Yu, Khalid Mehmood, Zhen Li, Shucheng Chang, Weiping Liu, Daniel Rosenfeld, Richard C. Flagan, John H. Seinfeld

**Affiliations:** 1 Research Center for Air Pollution and Health, Key Laboratory of Environmental Remediation and Ecological Health, Ministry of Education, College of Environmental and Resource Sciences, Zhejiang University, Hangzhou, Zhejiang, 310058 P.R. China; 2 Division of Chemistry and Chemical Engineering, California Institute of Technology, Pasadena, CA 91125 USA; 3 Institute of Earth Sciences, The Hebrew University of Jerusalem, Jerusalem, Israel

## Abstract

Widespread economic growth in China has led to increasing episodes of severe air pollution, especially in major urban areas. Thermal power plants represent a particularly important class of emissions. Here we present an evaluation of the predicted effectiveness of a series of recently proposed thermal power plant emission controls in the Beijing-Tianjin-Hebei (BTH) region on air quality over Beijing using the Community Multiscale Air Quality(CMAQ) atmospheric chemical transport model to predict CO, SO_2_, NO_2_, PM_2.5_, and PM_10_ levels. A baseline simulation of the hypothetical removal of all thermal power plants in the BTH region is predicted to lead to 38%, 23%, 23%, 24%, and 24% reductions in current annual mean levels of CO, SO_2_, NO_2_, PM_2.5_, and PM_10_ in Beijing, respectively. Similar percentage reductions are predicted in the major cities in the BTH region. Simulations of the air quality impact of six proposed thermal power plant emission reduction strategies over the BTH region provide an estimate of the potential improvement in air quality in the Beijing metropolitan area, as a function of the time of year.

## Introduction

Among the 500 largest cities of China, it is estimated that fewer than 1% can meet World Health Organization air quality guidelines (10 µg m^−3^ for annual mean and 25 µg m^−3^ for 24-hour mean for fine particulate matter with aerodynamic diameter <2.5 µm(PM_2.5_)). Moreover, several of these cities are among the most polluted cities in the world^1^. In Beijing, the capital and economic, cultural, and political center of China, the number of so-called *haze days* (with visibility <10 km) has increased intensively since 2011^2^. For example, in January 2013, 25 haze days were recorded, with two severe episodes occurring during 9–15 January and 25–31 January with maximum hourly PM_2.5_ mass concentrations of 680 and 530 µg^−3^, respectively^3^. Severe haze periods are characterized by intense secondary pollutant formation, stationary meteorological conditions, high relative humidity (RH), and low planetary boundary (PBL) depth^4^.

Of electric power capacity in China, 962 million KW in 2010, 80% was supplied by thermal power plants. Moreover, the thermal power plant sector in China is estimated to account for 31–59% of anthropogenic emissions of SO_2_^5–9^, 21–44% of NO_x_^10,11^, and 9% of primary PM^11^. In Beijing, SO_2_, NO_x_, and PM_10_ attributed to thermal power plants are estimated to account for approximately 49%, 27%, and 11% of total emissions in the Beijing area, respectively. Specifically, emissions from coal burning have been estimated to account for ~26% of total PM_2.5_ sources during the January 2013 severe haze episodes in Beijing^12^. Here, we evaluate the potential effects of emission control policies for thermal power plants on air quality in Beijing.

**Figure 1:**
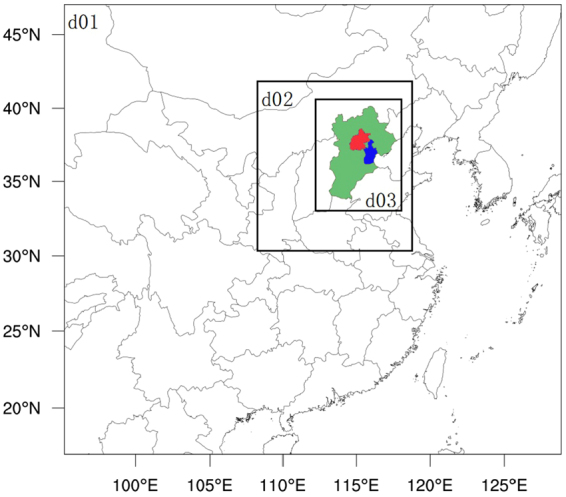
Triple nesting domains for computational implementation of the CMAQ model: d01 covers most of China and part of East Asia with the resolution of 36 × 36 km; d02 covers the Northern China Plain (NCP) with the resolution of 12 × 12 km; d03 covers the Beijing-Tianjin-Hebei (BTH) region with the resolution of 4× 4 km. Green, orange, and blue colors represent Heibei, Beijing, and Tianjin, respectively. The maps were created by NCAR Command Language (NCL) (http://www.ncl.ucar.edu/).

Comprehensive 3-D air quality models (see Methods) have been used to evaluate the impacts of emission control strategies on local and regional air quality in China^13–17^. Wang *et al*.^13^ assessed the air quality improvement resulting from targeted SO_2_ and NO_x_ emission controls for 2010; it was predicted that annual PM_2.5_ and SO_2_ could decline by 3–15 µg m^−3^ (4–25%) and 30–60%, respectively. Wang *et al*.^16^ predicted that for areas with PM_2.5_concentrations exceeding the second-level air quality standard (25 µg m^−3^ for annual mean and 50 µg m^−3^ for 24-hour mean) in 31 selected provinces in China, the annual mean SO_2_ and NO_x_ concentrations in 2020 relative to 2010 could be reduced by 40.0% and 31.6%, respectively, and the annual mean PM_2.5_ concentration could decrease by 17.2%. Here we present results of the application of the CMAQ air quality model (see Methods) to evaluate the effects of thermal power generation emission controls in the Beijing-Tianjin-Hebei (BTH) region (Fig. 1).

## Emission Control Scenarios

Table 1 lists the emission controls corresponding to a range of policies for thermal power plants in the BTH region. All thermal power plants in the BTH region use coal as the only fuel. These include the policy A “Emission standard of air pollutants of thermal power plants (GB13223-2011)” (released on July 29, 2011) and policy B “Action Plan for the Transformation and Upgrading of Coal Power Energy Conservation and Emission Reduction (2014–2020)” (released by the Chinese government on September 12, 2014) (http://www.sdpc.gov.cn/gzdt/201409/t20140919_626240.html). The eight emission scenarios in Table 1 will be evaluated here for thermal power plants in the BTH region. Since the policy A was carried out on January 1, 2013, it is assumed that the baseline scenario, Case 1, was implemented as of that date. Case 1 therefore serves as a basis: (1) to simulate current air quality in the BTH region for the base year; (2) to evaluate model performance; and (3) to determine the effectiveness of other potential policies to improve air quality in Beijing. According to policy A, key areas such as the BTH region need to conform to special emission limits. Key areas are defined as those with high land development density, weakened atmospheric carrying capacity, and fragile ecological environment. In Case 2 for special emission limits, the emission standards for SO_2_, smoke dust, and PM_2.5_ as listed in Table 1 are changed to 50, 20, and 10 mg m^−3^, respectively.

**Table 1:** Emission control standards of different cases for thermal power plants in the BTH region.

Scenario Cases	Description	Emission Standards (mg m^−3^)			
		SO_2_	NO_2_	Smoke Dust	PM_2.5_
1^a^	Baseline scenario A	200	100	30	15
2^a^	Special emission limits	50	100	20	10
3^b^	Ultra-low emissions B	35	50	10	8
4	Adjustment of structures	200	100	30	15
5^c^	Extremely ultra-low emissions	35	50	5	4
6^c^	“Near zero” emissions	15	25	1	0.8
7^c^	Green power generation	20	30	4.5	4
8	No thermal power plant emissions	0	0	0	0

^a^See http://kjs.mep.gov.cn/hjbhbz/bzwb/dqhjbh/dqgdwrywrwpfbz/201109/W020130125407916122018.pdf.

^b^See http://www.zhb.gov.cn/gkml/hbb/gwy/201409/t20140925_289556.htm.

^c^Cases 5, 6, 7 are carried out only for the month of January over the domain d02 with the grid resolution 12 km.

**Figure 2:**
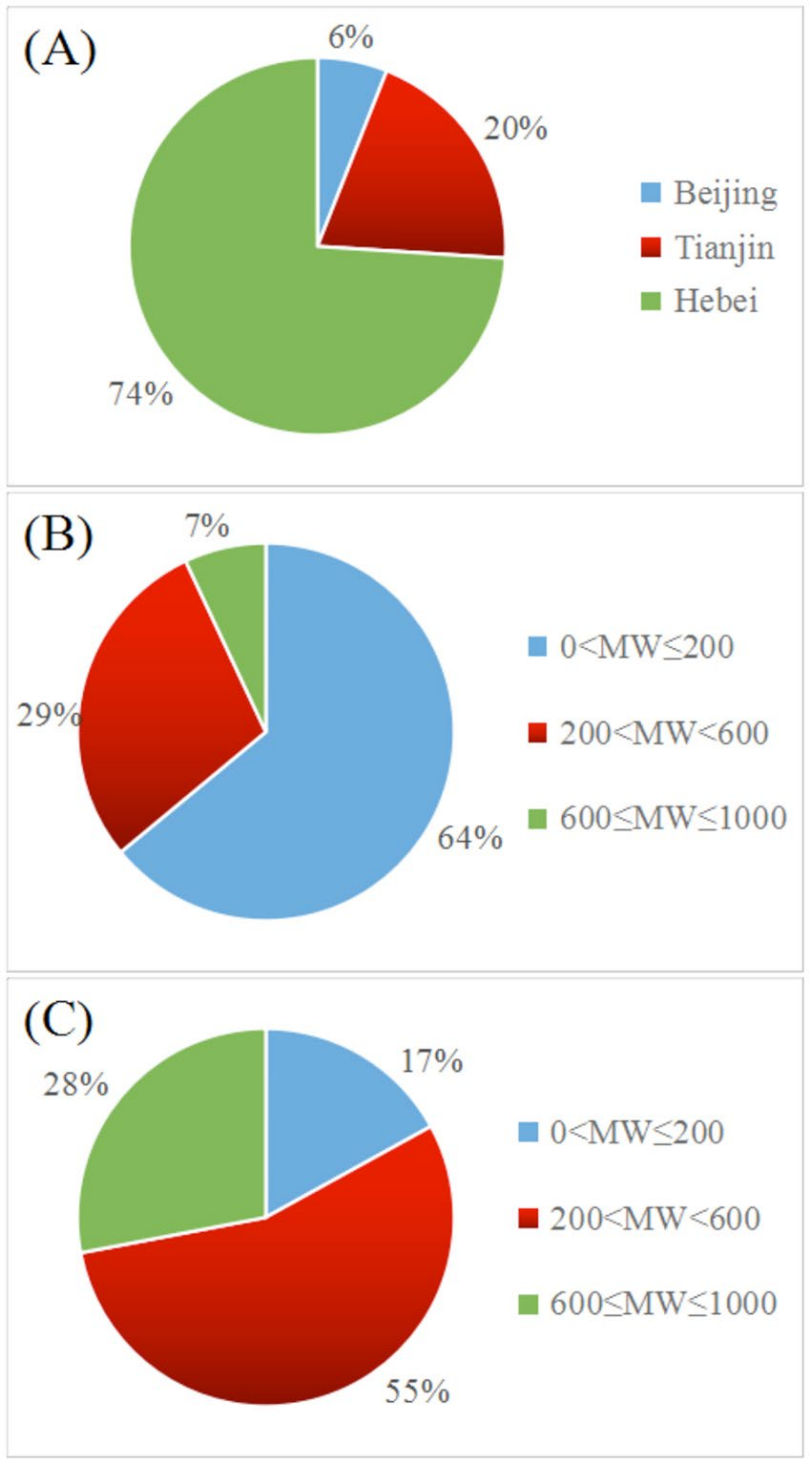
Proportional distributions of thermal power plants in the BTH region in 2013 based on (**A**) unit capacity for the three areas, (**B**) the distribution of units by MW capacity, and (**C**) the unit capacity for the entire region.

**Figure 3:**
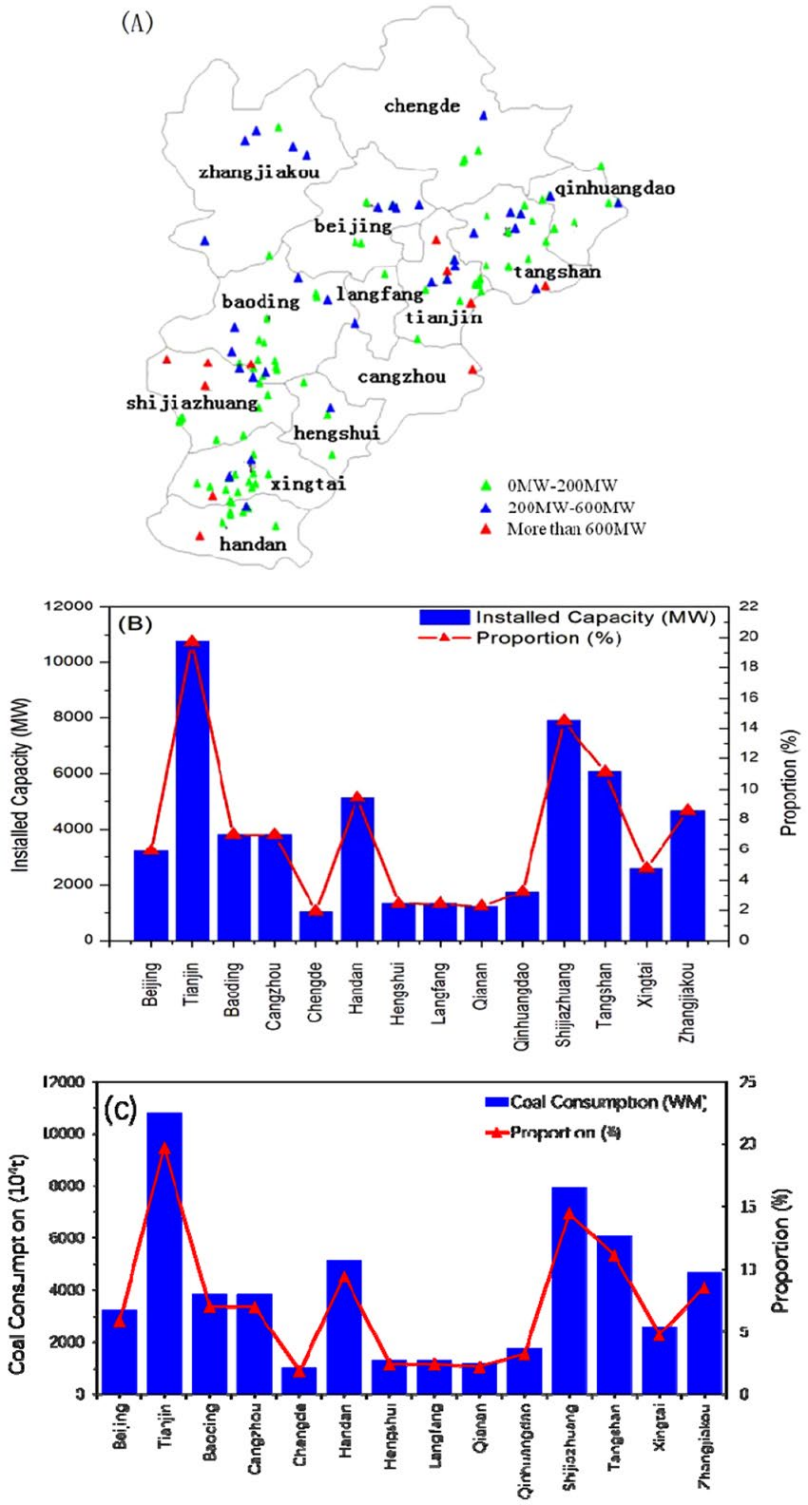
BTH region. (**A**) Locations of thermal power plants; (**B**) total capacity of thermal power plants and their distribution by city; (**C**) coal consumption distribution in the BTH region in 2013. The maps were created by NCAR Command Language (NCL) (http://www.ncl.ucar.edu/).

According to the policy B, all thermal power units must meet ultra-low emission standards. In Case 3, emission standards of SO_2_, NO_2_, smoke dust, and PM_2.5_ are 70%, 50%, 50%, and 80% of those of Case 2, respectively. Policy B includes acceleration of the upgrade of active thermal power units, especially for those <200 MW. Figure 2 shows the distribution and percentage of the thermal power plants in terms of unit capacities in the three areas for the BTH region in 2013 (http://ieimodel.org/jjjdqhdhypfqd). Units with capacity <200 MW and >600 MW account for 64% and 7% of the total units, respectively (Fig. 2B), while the unit capacity is mainly located in Tianjin, Shijiazhuang, Tangshan, and Handan, especially in the southwest and east of the BTH region (Fig. 3A and B). In Case 4, all units with output <200 MW are assumed to be eliminated to evaluate the extent to which this structural adjustment would affect Beijing air quality.

The Shenhua Group Corporation Ltd. proposed three emission control policies including “Extremely ultra-low” (Case 5), “Near zero” (Case 6), and “Green power generation” (Case 7) (http://www.shenhuagroup.com.cn/shjtww/1382682123426/201506/cc7d7362c2224378ab536a27aa30b8b7.shtml). The Shenhua Group is the largest coal supplier in the world. In Case 5, the emission standards for smoke dust and PM_2.5_ are set to be one-half, but remain unchanged for SO_2_ and NO_2_, relative to Case 3. In Case 6, emission standards of SO_2_, NO_2_, smoke dust, and PM_2.5_ are set at 40%, 50%, 20%, and 20% of those of Case 5, respectively. In Case 7,emission standards for SO_2_, NO_2_, smoke dust, and PM_2.5_ lie between those of Cases 5 and 6. Case 8 represents the hypothetical total absence of thermal power plants in the BTH region. Since air quality in Beijing is poorest in the winter, simulations for Cases 5, 6, and 7 are carried out only for the month of January over the domain d02 with the grid resolution of 12 km (Fig. 1).

### Predicted influence of emission control policies on Beijing air quality

**Figure 4:**
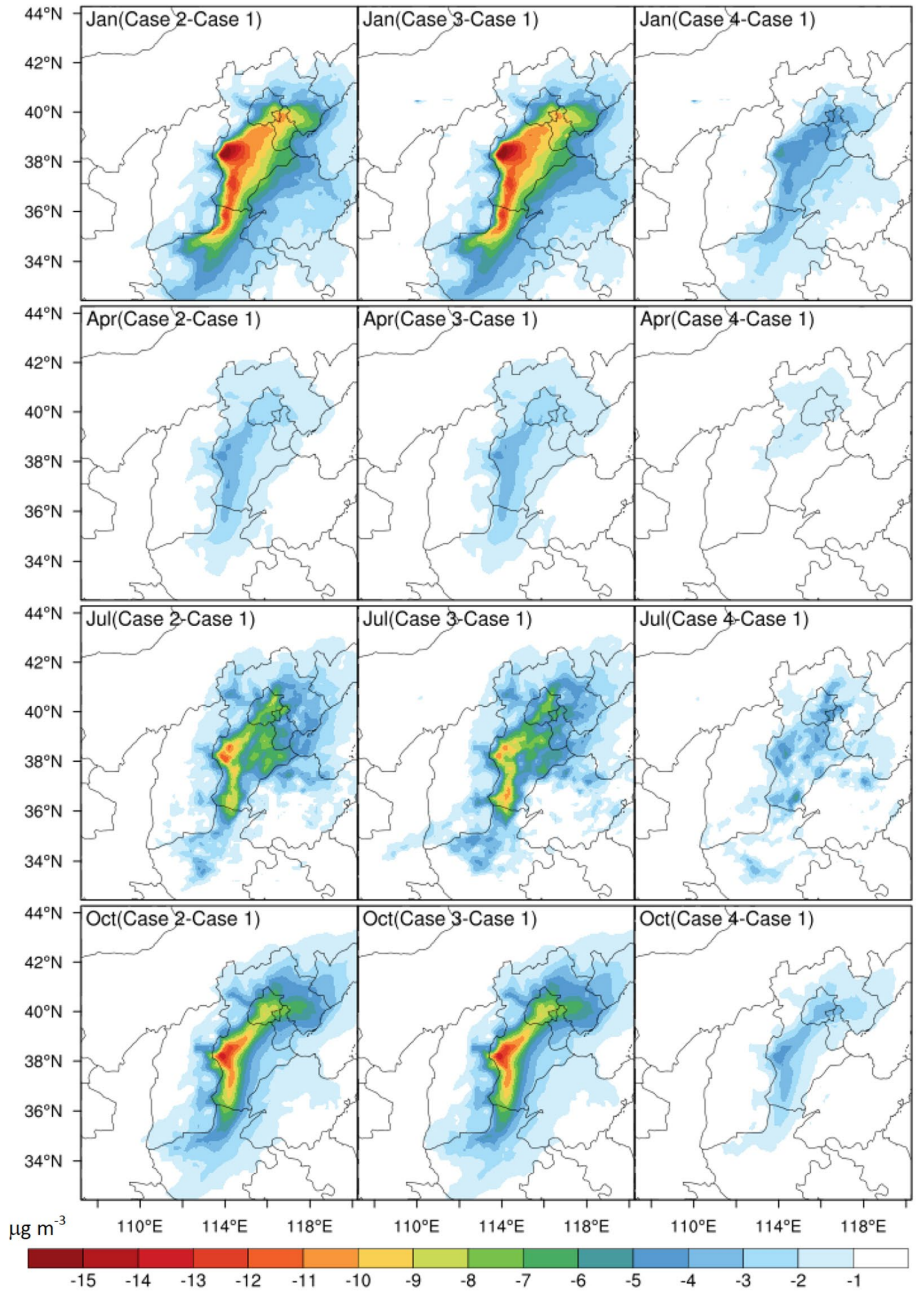
Distribution of PM_2.5_ reduction amount (µg m^−3^) in January, April, July, and October for Cases 2, 3, and 4 relative to the base Case 1 on the basis of the simulations with grid resolution of 12 km. The maps were created by NCAR Command Language (NCL) (http://www.ncl.ucar.edu/).

**Figure 5:**
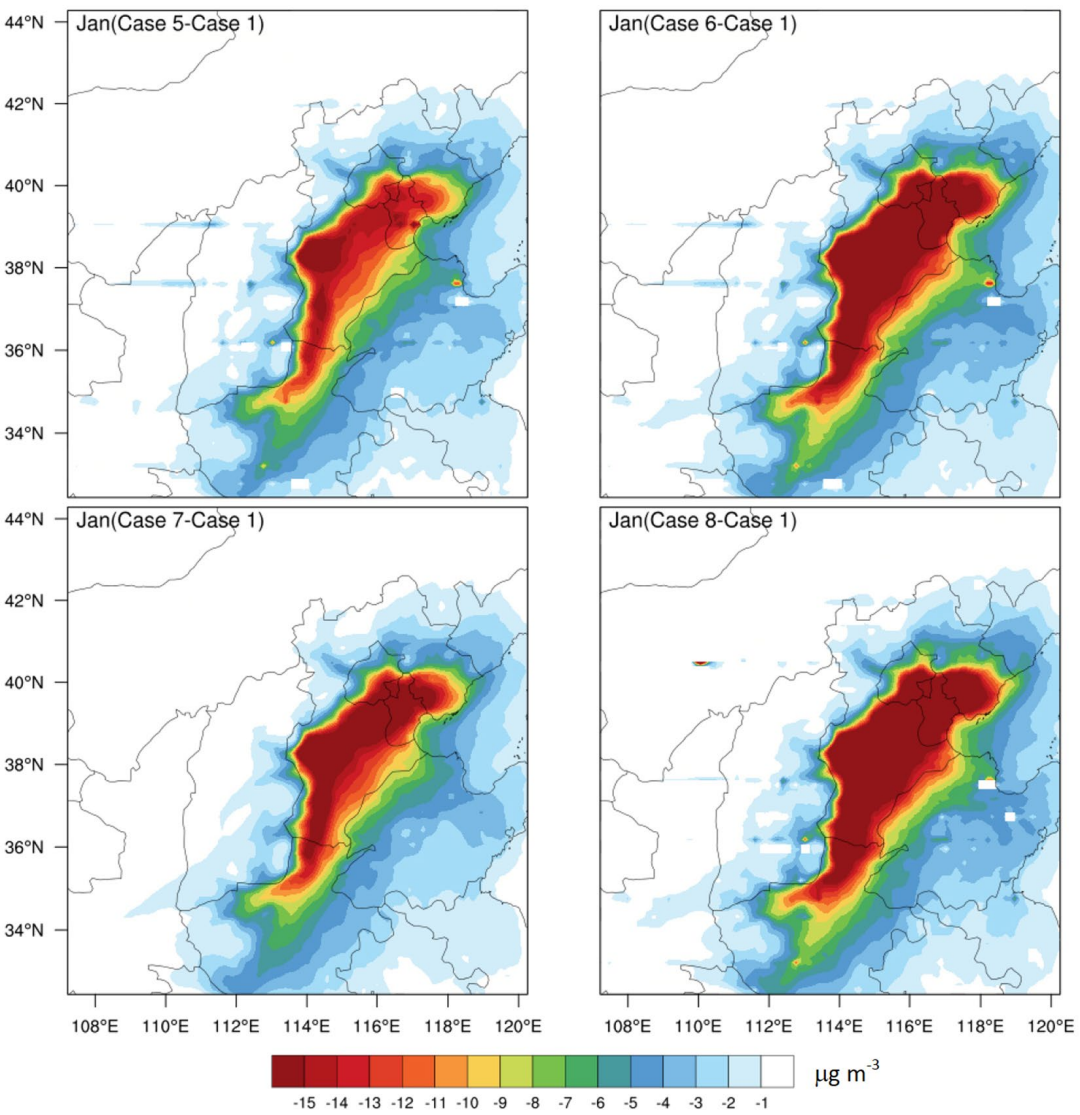
Distribution of predicted monthly mean reductions of PM_2.5_ (µg m^−3^) for Cases 5, 6, 7, and 8 relative to that of Case 1 in January 2013. The maps were created by NCAR Command Language (NCL) (http://www.ncl.ucar.edu/).

Monthly mean concentrations of PM_2.5_, PM_10_, NO_2_, CO, and SO_2_ for different emission scenarios at 12 monitoring stations in the Beijing are used as a basis to assess the predicted improvement in Beijing air quality associated with the emission control policies relative to those of the baseline emission control scenario (Case 1). Table 2 summarizes the predicted annual percentage reductions in Cases 2, 3, 4 relative to Case 1 (CO, SO_2_, NO_2_, PM_2.5_, and PM_10_), while Table 3 shows the percentage reductions for Cases 5, 6, 7 in January, and Table 4 shows the percent reductions for total removal of power plants. Spatial distributions of the reduction of PM_2.5_ for January, April, July, and October are shown in Fig. 4, while Fig. 5 shows the spatial distributions of predicted monthly mean reductions for PM_2.5_ for implementation of Cases 5, 6, 7 relative to that of Case 1. Cases 2 and 3 lead to higher reduction percentages than Case 4 for all species except CO, for which all three emission control policies predict similar reduction percentages (Table 2). Predicted monthly mean reductions in Beijing in January for Cases 5, 6, 7 for PM_2.5_ range from 9.05% to 12.12% (Table 3). The spatial distributions of predicted reduction of PM_2.5_ in different months for Cases 2, 3, 4, shown in Fig. 4, indicate that the largest percent reduction of PM_2.5_ would occur in January, especially in Tianjin and over the central part of Hebei (southwest of Beijing), for Cases 2 and 3. This response is consistent with the dominant locations of the coal-fired power plants (Fig. 3A).

**Table 2:** Predicted annual percentage reductions and amounts* (in parentheses) of Cases 2, 3, and 4 relative to Case 1 in Beijing with a resolution of 4 × 4 km.

Species	$\frac{{\bf{Case}}\,{\bf{2}}-{\bf{Case}}\,{\bf{1}}}{{\bf{Case}}\,{\bf{1}}}$	$\frac{{\bf{Case}}\,{\bf{3}}\mbox{--}{\bf{Case}}\,{\bf{1}}}{{\bf{Case}}\,{\bf{1}}}$	$\frac{{\bf{Case}}\,{\bf{4}}\mbox{--}{\bf{Case}}\,{\bf{1}}}{{\bf{Case}}\,{\bf{1}}}$
CO	−20.65 (−0.39)	–20.42 (–0.38)	–20.41 (–0.38)
SO_2_	−7.37 (−3.23)	−7.30 (−3.20)	−3.13 (−1.37)
NO_2_	−7.81 (−3.93)	−7.47 (−3.76)	−3.49 (−1.75)
PM_2.5_	–6.31 (−6.59)	−6.18 (−6.45)	−2.63 (−2.75)
PM_10_	−5.94 (−6.90)	−5.82 (−6.76)	−2.61 (−3.03)

^*^Units of CO are mg m^−3^ and those of other species are µg m^−3^.

**Table 3:** Predicted percentage reductions and amounts* (in parentheses) corresponding to Cases 5, 6, and 7 relative to Case 1 in January in Beijing.

Species	$\frac{{\bf{Case}}\,{\bf{5}}\mbox{--}{\bf{Case}}\,{\bf{1}}}{{\bf{Case}}\,{\bf{1}}}$	$\frac{{\bf{Case}}\,{\bf{6}}\,{\boldsymbol{\mbox{--}}}{\bf{Case\; 1}}}{{\bf{Case\; 1}}}$	$\frac{{\bf{Case}}{\bf{7}}\,{\boldsymbol{\mbox{--}}}{\bf{Case\; 1}}}{{\bf{Case\; 1}}}$
CO	−22.98 (−0.60)	−27.73 (−0.73)	−23.26 (−0.61)
SO_2_	−9.21 (−4.51)	−14.82 (−7.26)	−13.66 (−6.68)
NO_2_	−8.89 (−5.70)	−11.10 (−7.11)	−9.29 (−5.95)
PM_2.5_	−9.05 (−13.76)	−12.12 (−18.43)	−10.17 (−15.46)
PM_10_	−8.62 (−13.87)	−11.75 (−18.71)	−10.61 (−17.07)

^*^Units of CO are mg m^−3^ and those of other species are µg m^−3^.

**Table 4:** Percent contributions of thermal power plant emissions in the BTH region to atmospheric species in Beijing based on assumed total removal of power plants (Case 8).

Species	CO	SO_2_	NO_2_	PM_2.5_	PM_10_
Jan	−39.42	−23.55	−22.02	−27.07	−26.59
Apr	−34.12	−22.20	−22.99	−21.03	−21.82
Jul	−35.16	−23.43	−21.63	−22.19	−20.97
Oct	−38.73	−22.97	−24.73	−22.16	−24.16
Annual	−37.63	−23.09	−23.02	−23.78	−24.04

To meet increasingly stringent emission standards and obtain maximum emission reductions from the coal-fired power plants, “Extremely ultra-low” (Case 5), “Near zero” (Case 6), and “Green power generation” (Case 7) policies have been proposed. Emission levels from coal-fired power plants adopting these newly-designed emission control technologies are comparable with those from natural gas-fired plants. Table 3 summarizes predicted reduction percentages and amounts of PM_2.5_, PM_10_, NO_2_, CO, and SO_2_ in Beijing in January for these cases. Spatial distributions of PM_2.5_ reduction amounts over the BTH region in Fig. 5 show that most of PM_2.5_ reduction amounts are located in the southeast Beijing, Tianjin and the central Hebei with the largest reductions from Cases 6 (“Near zero emission”) and 8 (“No thermal power plants”) and lowest reductions from Case 5 (“Extremely ultra-low”), being consistent with the emission control standards (Table 1) and domain locations of the coal-fired power plants (Fig. 3A), as expected.

Predicted SO_2_ reduction percentages for the newly-designed emission control policies (Cases 5, 6, 7) are predicted to range from −9.2% to −14.8%, exceeding those of the current emission control policies (ranging from −2.7% to −6.0%). As expected, among these emission policies, Case 6 (“Near zero emission”) leads to the largest reduction percentages for all species (PM_2.5_, PM_10_, NO_2_, CO, and SO_2_) (Table 3), consistent with the emission control standards in Table 1. For Case 8 “No thermal power plants” in Table 4, the predicted annual contributions of thermal power plants over the BTH region to the concentrations of CO, SO_2_, NO_2_, PM_2.5_, and PM_10_ in Beijing are 37.6%, 23.1%, 23.0%, 23.8% and 24.0%, respectively. Thermal power plants over the BTH region are predicted to contribute appreciably to CO concentrations in Beijing (Table 4). The lowest values in Beijing contributed by thermal power plants over the BTH region are predicted to occur in April for CO, SO_2_, and PM_2.5_, whereas in July for NO_2_ and PM_10_.

In comparison with the prediction of the current emission control policies (Cases 2, 3 and 4) in Table S2, Cases 5, 6 and 7 lead to further modest reductions of PM_2.5_, PM_10_, NO_2_, CO and SO_2_ in Beijing (see Table S3). For example, the reduction percentages for PM_2.5_ for the newly-designed emission control policies lie between −9.1% and −12.1%, about 10% to 7% higher than those of the current emission control policies (range from −2.0% to −5.1%) in January Predictions for PM_10_ are close to those for PM_2.5_. CO reduction percentages for the newly-designed emission control policies range from −23.0% and −27.7%, about 3% to 7% higher than those of the current emission control policies (range from −20.4% to −20.8%), and for SO_2_, reduction percentages for the newly-designed emission control policies range from −9.2% and −14.8%, about 6% to 12% higher than those of the current emission control policies (range from −2.7% to −6.0%). As expected, among these newly-designed emission policies, Case 6 (“Near zero emission”) predicts the largest reduction percentages for all species (PM_2.5_, PM_10_, NO_2_,CO and SO_2_), followed by Cases 7 and 5 (see Table S3), consistent with the emission control standards in Table 1. The results in Tables S2 and S3 suggest that the extent to which it is worth carrying out these newly-designed emission control policies depends on their economics.

Tables S10–S21 (Supplemental Information) summarize predicted annual PM_2.5_, CO, SO_2_, NO_2_, and PM_10_ reduction percentages in 12 other major Chinese cities (Tianjin, Baoding, Cangzhou, Chengde, Handan, Hengshui, Qinhuangdao, Shijiazhuang, Tangshan, Xingtai, Zhangjiakou, Langfang) over the BTH region for Cases 2, 3, 4 on the basis of simulations at the 12 km grid resolution. The annual mean predicted reduction percentages for PM_2.5_ are between −4.5% and −10.7%, with the highest value in Zhangjiakou and the lowest value in Hengshui for Cases 2 and 3, while they lie between −1.7% and −3.4% for Case 4. Predicted reduction percentages for Case 3 are slightly higher than those of Case 2 for all species (CO, SO_2_, NO_2_, PM_2.5_, and PM_10_) for all cities except Tianjin. Predicted monthly mean reduction amounts in January are the highest for CO, SO_2_, NO_2_, PM_2.5_, and PM_10_ for all three emission control policies, as expected.

## Conclusions

Thermal power plants (entirely coal-burning) are a major source of atmospheric emissions in China, controls on which are crucial for the improvement of air quality. In this work, we assess the potential air quality benefits in Beijing from different thermal power emission control policies for the BTH region. Predicted annual mean reduction percentages in Beijing lie between −5.3% and −6.3% for PM_2.5_, PM_10_, NO_2_, and SO_2_ for Cases 2 (“Special emissions limits”) and 3 (“Ultra-low emission standards”), and between −2.2% and −3.0% for Case 4 (“Adjustment of structures”), reflecting the effects of eliminating all units <200 MW. All three emission control policies are predicted to lead to similar reduction percentages between −18.9% and −20.7% for CO. In comparison with current emission control policies, the newly-designed control policies considered here are predicted to lead to reductions in January levels in Beijing between −8.6% and −14.8% for PM_2.5_, PM_10_, NO_2_, and SO_2_ and between −23.0% and −27.7% for CO. Predictions for Case 8 (“No thermal power plants”) in the BTH region suggest that the annual mean contributions of thermal power plants to the concentrations of CO, SO_2_, NO_2_, PM_2.5_, and PM_10_ in Beijing are 37.6%, 23.1%, 23.0%, 23.8%, and 24.0%, respectively, with the highest values occurring in January for all species except NO_2_. Predictions for the other 12 major cities over the BTH region for these emission control policies exhibit similar responses to those in Beijing.

## Methods

### Emission inventory

Anthropogenic emissions of SO_2_, NO_x_, CO, NMVOC, NH_3_, PM_10_ and PM_2.5_ over China are based on the Multi-resolution Emission Inventory for China (MEIC)^9^ for 2012 (www.meicmodel.org), while those for the rest of the domain were estimated on the basis of Emissions Database for Global Atmospheric Research (EDGAR): HTAP V2 (0.1° × 0.1°). Multi-resolution Emission Inventory for China (MEIC) is a dynamic technology-based inventory for more than 700 anthropogenic emitting sources developed for China covering the years from 1990 to 2013 by Tsinghua University following the work of INTEX-B^9^. With the detailed source classification by representing emission characteristics of different sectors, fuels, products, emission control and combustion/process technologies, the MEIC model can derive emissions which were aggregated to five sectors: power plants, industries, residential, transportation, and agriculture^9^. For example, transportation emissions at high spatial resolution were derived on the basis of vehicle population and emission factors, while the emissions at high-resolution model grids can be derived on the basis of a digital road map and weighting factors of kilometers traveled and road types^9^. The lumped speciated NMVOC emissions were derived for each source sector by allocating the total NMVOC emissions according to the speciation assignment approaches for different chemical mechanisms such as CB05 in the MEIC. Temporal variations and gridded emissions were created for each sector using different temporal profiles and spatial aggregations.

In this work, power plant emissions in the BTH region generated by the Appraisal Center for Environment and Engineering in the State Environmental Protection Ministry of China (http://ieimodel.org/jjjdqhdhypfqd) were used to replace those in the MEIC emission inventory. These new power plant emissions in the BTH region were derived on the basis of the power plant emission data information from CEMS (Continuous Emission Monitoring System) measurements, Environmental Impact Assessment (EIA) measurements and follow-up inspection for the power plants in China (http://ieimodel.org/jjjdqhdhypfqd). Power plant emissions were estimated for each thermal power plant unit on the basis of fuel consumption rates, fuel quality, combustion technology and emission control technology.

### Model description

This study employs the Weather Research and Forecast (WRF, version 3.4)^17^ model coupled with Community Multiscale Air Quality (CMAQ, version 5.0) model^18–22^. We use a nested grid configuration with an outer grid encompassing most of China and part of eastern Asia (36 km grid resolution), the first inner grid encompassing the North China Plain (12 km grid resolution) and the second inner grid covering the BTH region (4 km grid resolution) (Fig. 1). The physics package of the WRF3.4 (ARW) includes version 2 of the Kain-Fritsch cumulus cloud parameterization (KF2)^23^, Morrison *et al*. two-moment cloud microphysics^24–26^, the Asymmetric Convective Model version 2 (ACM2) for the planetary boundary layer (PBL)^27,28^, the RRTMG radiation mechanism and the Pleim-Xiu land-surface model^29,30^, with indirect soil moisture and temperature nudging^31,32^. The aerosol module of the CMAQ model is AERO6, and the gas-phase chemical mechanism is CB05. Boundary conditions for the inner domains are derived from simulations of the outer domains and the meteorological initial, and lateral boundary conditions for the outermost domain were derived from the National Center for Environmental Prediction (NCEP) final analysis dataset with a spatial resolution of 1° × 1° and a temporal resolution of 6 h. The default chemical boundary conditions (BCONs) in the CMAQ model were used in the simulations for the outermost domain of base year 2013. The WRF-CMAQ model simulation periods include January, April, July, and October in 2013 to represent winter, spring, summer, and autumn seasons, respectively.

### Model performance for PM_2.5_, PM_10_, NO_2_, SO_2_, and CO

**Figure 6:**
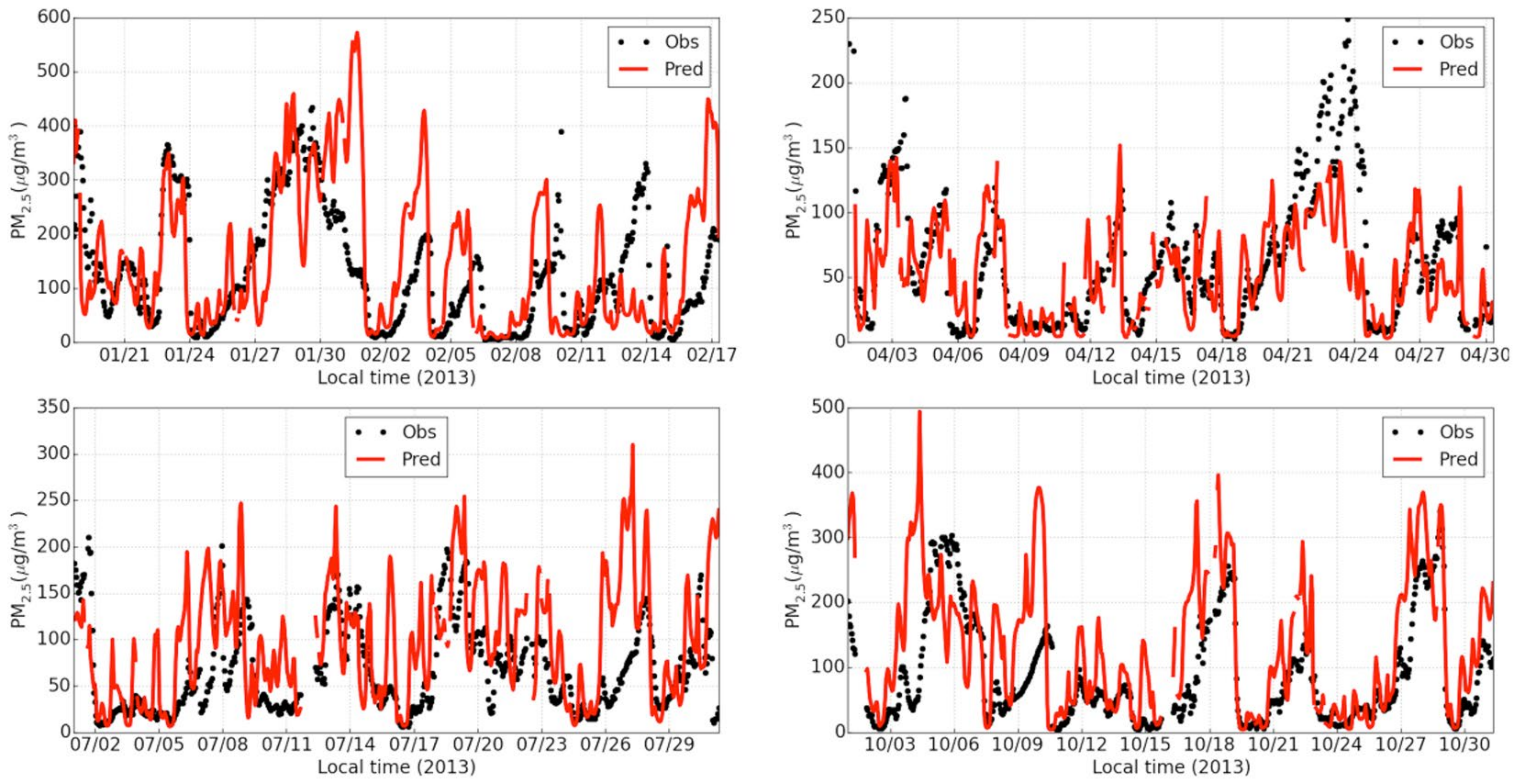
Time-series comparisons of the observed (Obs) and predicted (Pred) hourly mean PM_2.5_ concentrations in Beijing for January-February, April, July, and October 2013.

To evaluate model performance, the mean bias (MB), normalized MB (NMB) and root mean square error (RSME), normalized mean error (NME) and correlation coefficient (r) are calculated^33^. Monthly and annual results of model performance evaluation for PM_2.5_, PM_10_, NO_2_, CO, and SO_2_ in Beijing are summarized in Table S1 (see Supplemental Information) for the baseline emission scenario (Case 1) at the grid resolution of 12 km. Figure 6 shows the time-series comparisons of the observed and predicted hourly mean PM_2.5_ concentrations in Beijing for each month at the grid resolution of 12 km. Model performance for PM_2.5_, PM_10_, NO_2_, CO, and SO_2_ in Beijing is similar for the simulations at grid resolutions of 36, 12, and 4 km (see Supplemental Information). For example, the NMB values for PM_2.5_ are 19.6%, 26.6%, and 23.2% at 36 km, 12 km, and 4 km grid resolutions, respectively, on the basis of the annual simulations, while the corresponding NMB values for PM_10_ are 3.6%, 10.9%, and 8.1%, respectively.

Simulations at all three grid resolutions for SO_2_, CO, and PM_2.5_ exhibit poorer performance for July and October as compared with that for January and April (see Supplemental Information). Model simulations at all three grid resolutions (4 × 4, 12 × 12, 36 × 36 km) exhibit good performance for NO_2_ for all months except July. Significant overestimation of SO_2_in July is likely a result of non-representative locations and elevations of surface observation sites^34^, where SO_2_ is primarily emitted from stacks above local shallow inversion layers, while measurement stations are located close to the surface. Time-series comparisons of observed and predicted PM_2.5_ for different months in Beijing (Fig. 6) indicate that the predictions capture the hourly variations and broad synoptic changes in the observed PM_2.5_ concentrations.

Hourly observed concentrations (PM_2.5_, PM_10_, NO_2_, CO, and SO_2_) at 12 monitoring stations in Beijing obtained from the website “China’s air quality on-line monitoring analysis platform (http://www.aqistudy.cn/)” were used for evaluating the two-way coupled WRF-CMAQ model. The 12 Beijing monitoring stations include Wanshouxigong (38.87°N, 116.37°E), Changpingzhen (40.20°N, 116.23°E), Nongzhanguan (39.97°N, 116.47°E), Tiantan (39.87°N, 116.43°E), Guanyuan (39.94°N, 116.36°E), Haidianquwanliu (39.99°N, 116.32°E), Dongsi (39.95°N, 116.43°E), Gucheng (39.93°N, 116.23°E), Shunyixincheng (40.14°N, 116.72°E), and Aotizhongxin (40.00°N, 116.41°E). Since observational data before 18:00 January 17, 2013 are unavailable, the period from January 18 to February 17 of 2013 was used to represent January 2013. To evaluate model performance, concurrent hourly predicted concentrations at the monitoring sites were averaged in parallel with the hourly observations.

## Electronic supplementary material

Supplementary Information
