# Supplementary material for: Predicted impact of thermal power generation emission control measures in the Beijing-Tianjin-Hebei region on air pollution over Beijing, China
Source: Sci Rep. 2018 Jan 17;8:934. doi: 10.1038/s41598-018-19481-0 (PMC5772530; doi:10.1038/s41598-018-19481-0)
Supplement: Supplementary file 1 — Supplementary Information [file 41598_2018_19481_MOESM1_ESM.doc]

**Supplemental Information**

**Predicted impact of thermal power generation emission control measures in the Beijing-Tianjin-Hebei region on air pollution over Beijing, China**

Liqiang Wang^1+^，Pengfei Li^1+^, Shaocai Yu^1,2*^, Khalid Mehmood^1^, Zhen Li^1^, Shucheng Chang^1^, Weiping Liu^1^, Daniel Rosenfeld^3^, Richard C. Flagan^2^, and John H. Seinfeld^2*^

^1^Research Center for Air Pollution and Health; Key Laboratory of Environmental Remediation and Ecological Health, Ministry of Education, **C**ollege of Environmental and Resource Sciences, Zhejiang University, Hangzhou, Zhejiang 310058, P.R. China.

^2^Division of Chemistry and Chemical Engineering, California Institute of Technology, Pasadena, CA 91125, USA

^3^Institute of Earth Sciences, Hebrew University of Jerusalem, Jerusalem 9190401, Israel.

^+^These authors contributed equally to this work.

^*^Correspondence and requests for materials should be addressed to S.Y. (email: [shaocaiyu@zju.edu.cn](mailto:shaocaiyu@zju.edu.cn) or [shaocaiy@caltech.edu](mailto:shaocaiy@caltech.edu)) or J.S. (email: seinfeld@caltech.edu)

**Model performance for PM_2.5_, PM_10_, NO_2_, SO_2_, and CO.** Monthly and annual results of model performance evaluation for PM_2.5_, PM_10_, NO_2_, CO and SO_2_ in Beijing are summarized in Table S1 for the baseline emission scenario (Case 1) at the grid resolution of 12 km. Model performance evaluation and time-series comparisons for the model simulations at 36 km and 4 km resolution are summarized in Tables S6, S7 and shown in Figs. S1 and S2, respectively. As can be seen, model performance for PM_2.5_, PM_10_, NO_2_, CO and SO_2_ in Beijing is similar for the simulations at grid resolutions of 36, 12 and 4 km. For example, the NMB values for PM_2.5_ are 19.6, 26.6 and 21.2% at 36, 12 and 4 km grid resolutions, respectively, on the basis of the annual simulations, while the corresponding NMB values for PM_10_ are 3.6, 10.9 and 8.1%, respectively. Because of similar performance and results at the different grid resolutions, we present only the results of the model simulations at the 12 km grid resolution in the text, while the results from the simulations at the 36 and 4 km grid resolutions are in the Supporting Information for reference. The results in Tables S1, S6 and S7, and Figs. S1 and S2 demonstrate a good skill of the model in reproducing all pollutant (PM_2.5_, PM_10_, NO_2_ and CO) concentrations in Beijing for the four simulation months for the baseline emission scenarios except SO_2_ in July and October in which the NMB values for SO_2_ exceed 200%.

**Predicted influence of current emission control policies on Beijing air quality.** Table S2 summarizes the reduction amounts and percentages (PM_2.5_, PM_10_, NO_2_, CO and SO_2_) for the four simulation months and entire year. Cases 2 and 3 lead to higher reduction percentages than Case 4 in the four simulation months for all species except CO, for which all three emission control policies predict similar reduction percentages ranging from -18.9% to -20.7% (see Table S2). The results on the basis of simulations at 36 and 4 km grid resolutions are shown in Tables S8 and S9, and Figs. S3, S4, S5 and S6. Table S3 summarized predicted reduction percentages and amounts of PM_2.5_, PM_10_, NO_2_, CO and SO_2_ in Beijing for January for these cases. As expected, among these newly-designed emission policies, Case 6 (“Near zero emission”) predicts the largest reduction percentages for all species (PM_2.5_, PM_10_, NO_2_, CO and SO_2_), followed by Cases 7 and 5 (see Table S3), consistent with the emission control standards in Table 1. The results in Tables S1 and S2 suggest that the extent to which it is worth carrying out these newly-designed emission control policies depends on their economics. The results for the Case 8 “No thermal power plants” in Table S4 show that the annual contributions of thermal power plants over the BTH region to the concentrations of CO, SO_2_, NO_2_, PM_2.5_ and PM_10_ in Beijing are 37.6%, 23.1%, 23.0%, 23.8% and 24.0%, respectively, with the highest values in January for all species except NO_2_. Results in Tables S1, S2 and S3 also indicate that the thermal power plants over the BTH region make a large contribution (34.1 to 39.4%) to CO concentrations in Beijing, especially for units with capacity less than 200 MW which contribute to ~20% of CO in Beijing (Case 4 in Table S2). The lowest values in Beijing contributed by the thermal power plants over the BTH region are predicted to occur in April for CO, SO_2_ and PM_2.5_, while in July for NO_2_ and PM_10_, as shown in Table S4. This may be a result of different relative emission sources and meteorological dispersion conditions in April and July. Table S5 summarizes predicted PM_2.5_ reduction percentages in 12 other major cities (Tianjin, Baoding, Cangzhou, Chengde, Handan, Hengshui, Qinhuangdao, Shijiazhuang, Tangshan, Xingtai, Zhangjiakou, Langfang) over the BTH region for the current emission control policies (Cases 2, 3 and 4) for different seasons on the basis of simulations at the 12 km grid resolution. Predictions for other species (CO, SO_2_, NO_2_ and PM_10_) are presented in Table S10-S21. In comparison with those in Beijing, similar conclusions are obtained for the other 12 major cities. The annual mean predicted reduction percentage for PM_2.5_ are between -4.5% and -10.7%, with the highest value in Zhangjiakou and the lowest value in Hengshui for Cases 2 and 3, while they lie between -1.7% and -3.4% for Case 4. Predicted reduction percentages for Case 3 are slightly higher than those of the Case 2 for all species (CO, SO_2_, NO_2_, PM_2.5_ and PM_10_) for all cities except Tianjin, as shown in Tables S5 and S10-S21. For SO_2_, the largest annual reduction percentages are predicted to occur in Zhangjiakou (-19.8% to -21.0%) with the lowest values (-6.9% to -9.0%) in Baoding for Cases 2 and 3. For NO_2_, the largest annual reduction percentages are predicted in Zhangjiakou (-37.2% to -37.8%) with the lowest values (-7.5% to -8.8%) in Baoding for Cases 2 and 3. Predicted monthly mean reduction amounts in January are the highest for CO, SO_2_, NO_2_, PM_2.5_ and PM_10_ for all three emission control policies, as expected.

**
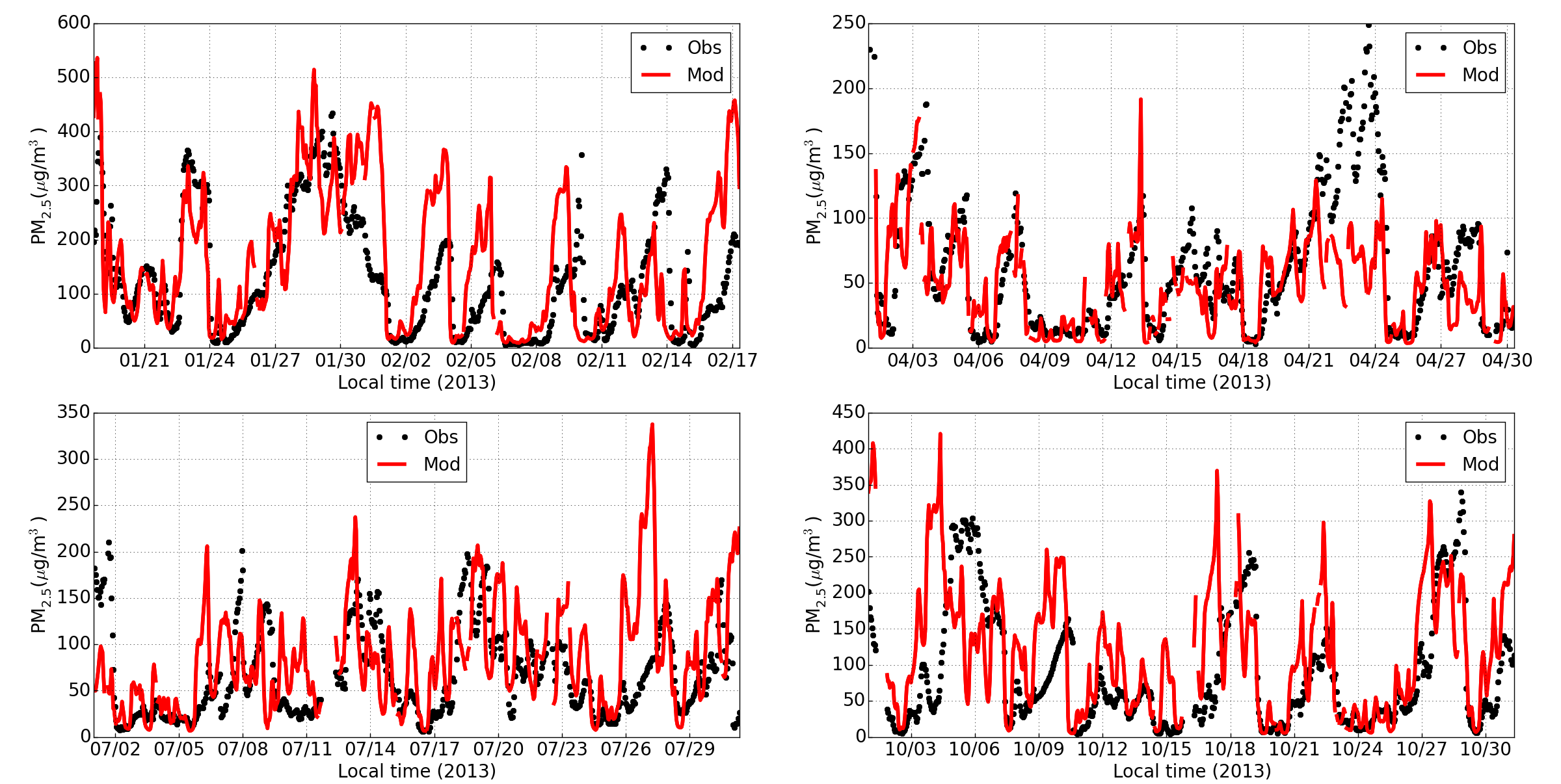
**

**Figure S1.** Time-series comparison of hourly observed and predicted PM_2.5_ concentrations in Beijing for the domain with 36-km resolution.

**
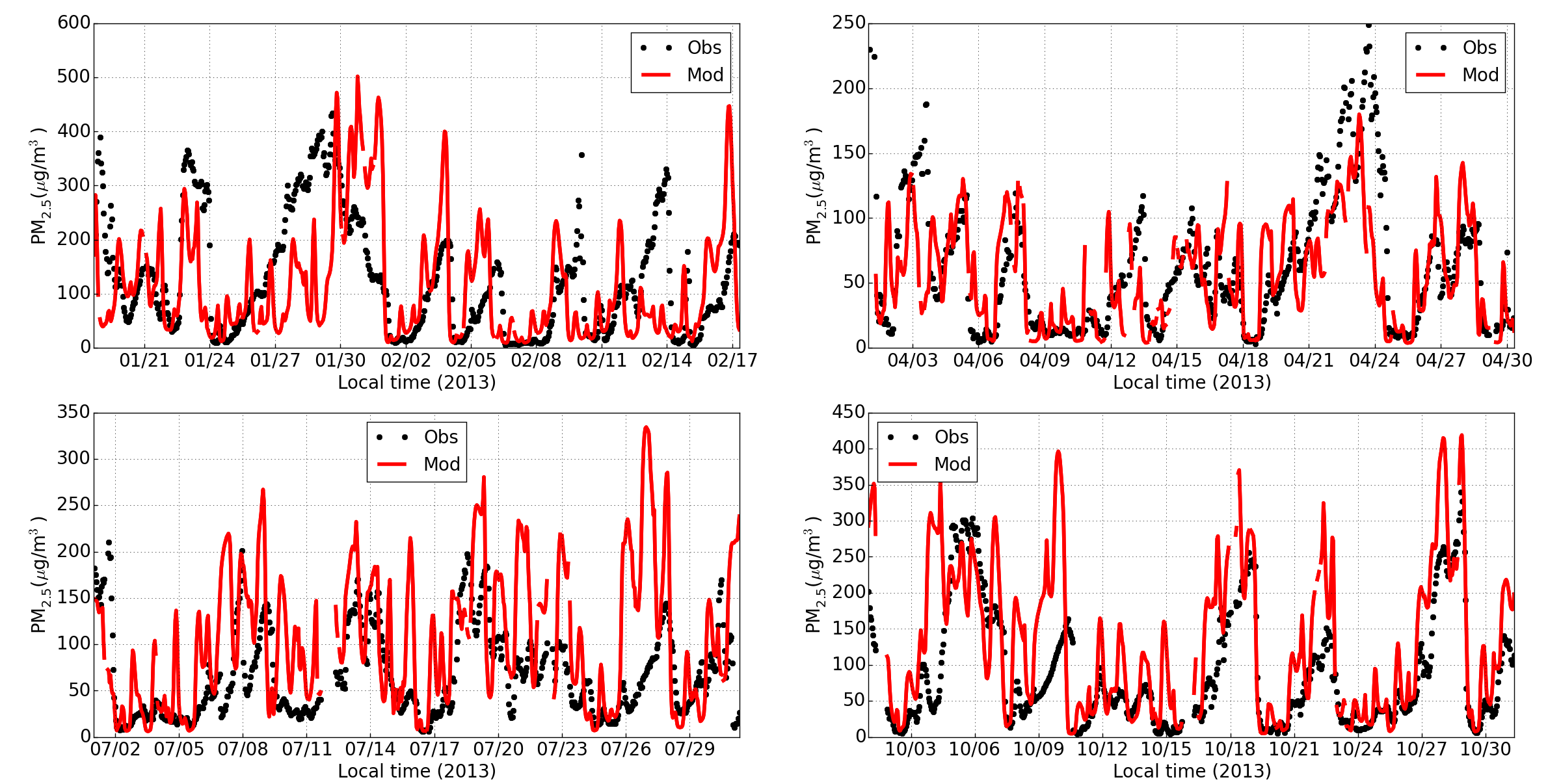
**

**Figure S2.** The same as Fig. S1 but for the domain with 4-km resolution.

**
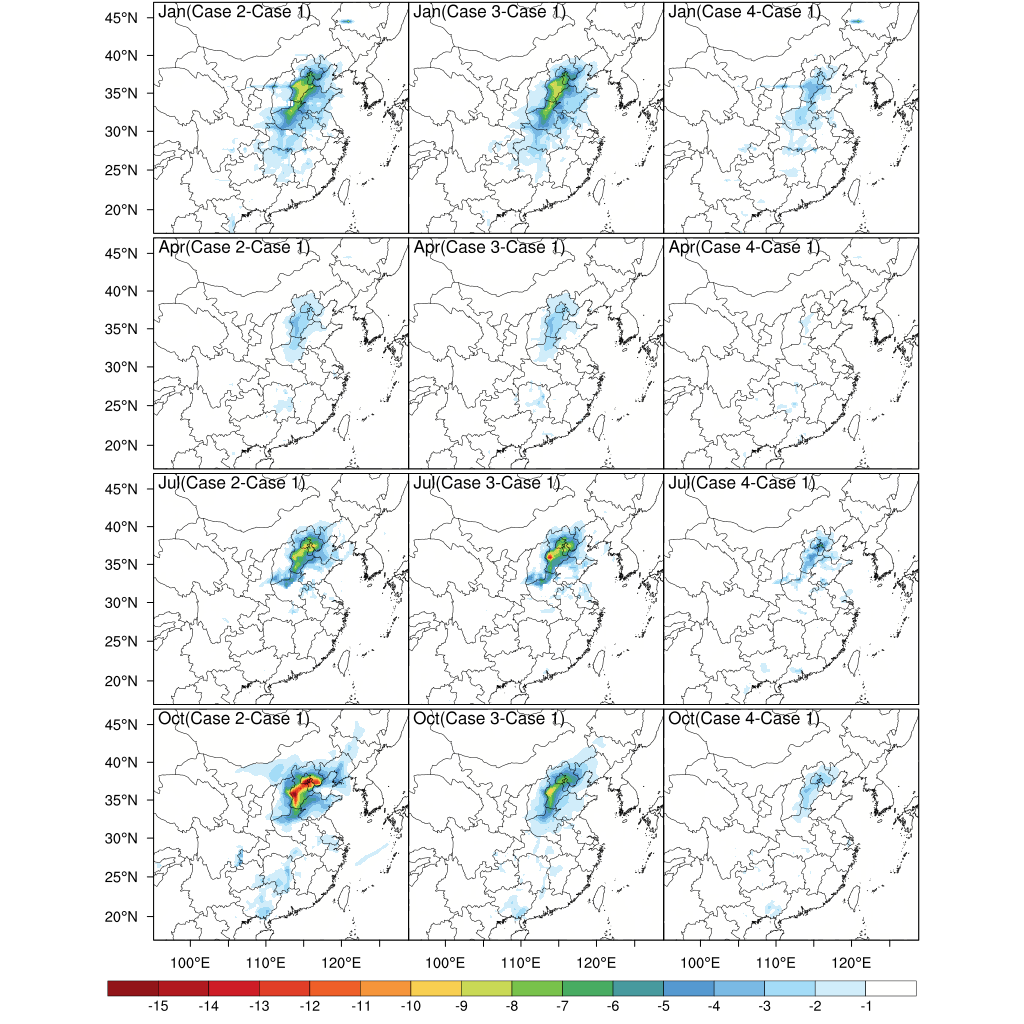
**

**Figure S3.** Distribution of monthly mean reduction (μg m^-3^) of PM_2.5_ in different months for the Cases 2, 3 and 4 relative to the base Case 1 on the basis of simulations with grid resolution of 36 km. The maps were created by NCAR Command Language (NCL) (http://www.ncl.ucar.edu/).

**
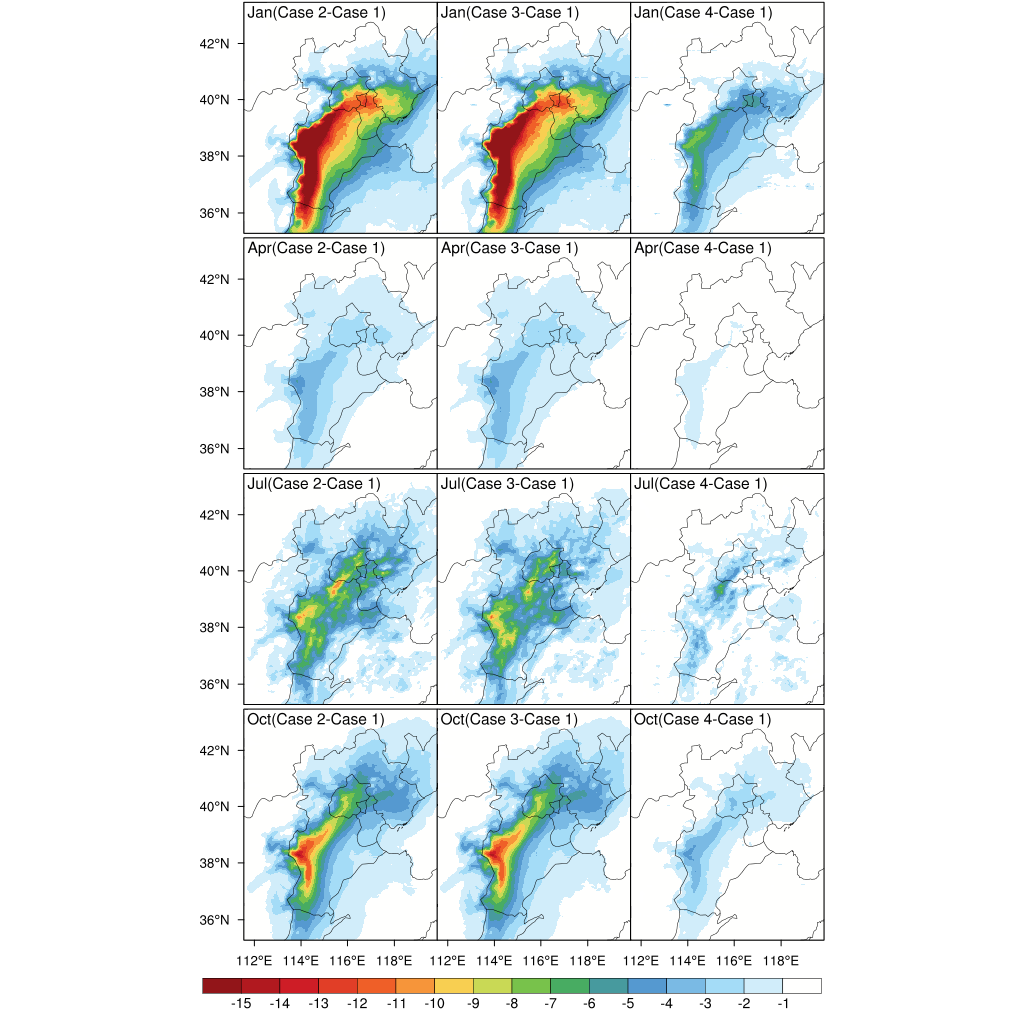
**

**Figure S4.** Distribution of monthly mean reduction (μg m^-3^) of PM_2.5_ in different months for the Cases 2, 3 and 4 relative to base Case 1 on the basis of simulations with grid resolution of 4 km. The maps were created by NCAR Command Language (NCL) (http://www.ncl.ucar.edu/).

**
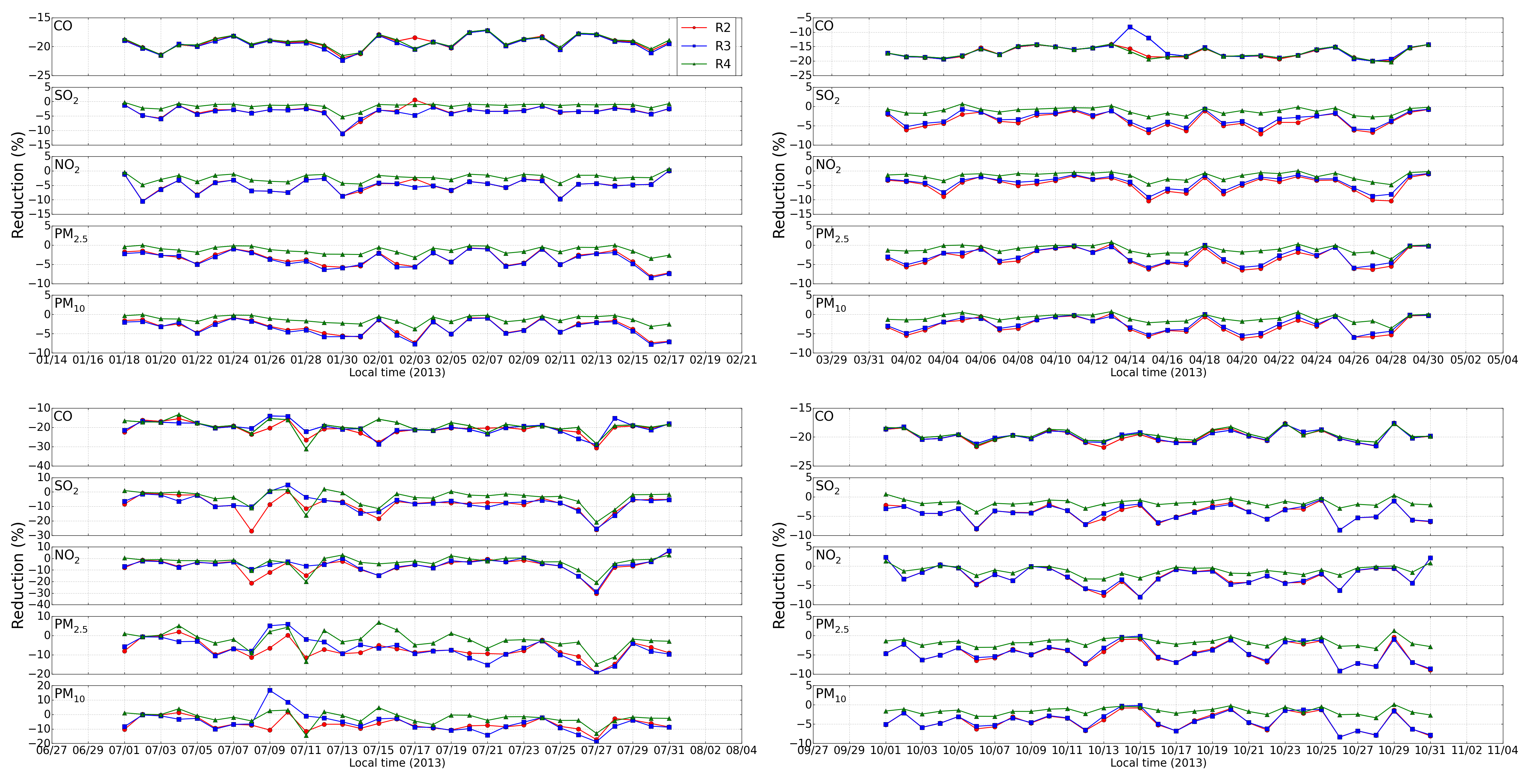
**

**Figure S5.** Time-series of daily mean reduction percentages of air pollutants of current cases in Beijing in different months with grid resolution of 36×36 km. R2, R3 and R4 represent (Case 2-Case 1)/Case 1, (Case 3-Case 1)/Case 1 and (Case 4-Case 1)/Case 1, respectively.


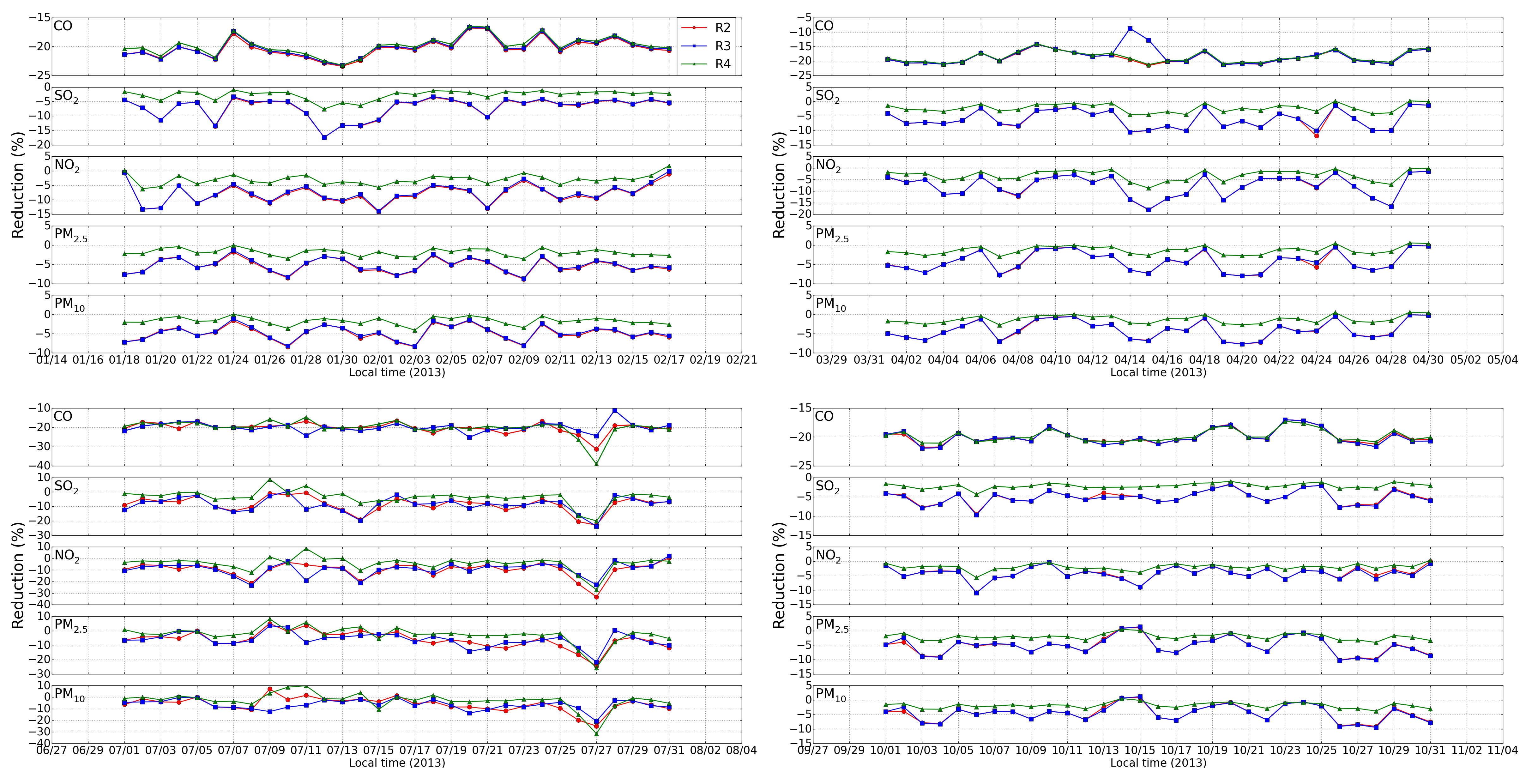


**Figure S6.** The same as Fig. S5 but for the model simulations at 4 km grid resolution.

**Table S1.** Evaluation of model performance in Beijing (Resolution: 12×12 km).

| Species |  | Obs | Mod | R | MB | NMB(%) | ME | NME(%) | N |
| --- | --- | --- | --- | --- | --- | --- | --- | --- | --- |
| CO | Jan | 2.54 | 2.56 | 0.65 | 0.02 | 0.84 | 0.88 | 34.21 | 713 |
|  | Apr | 0.82 | 0.92 | 0.57 | 0.10 | 11.86 | 0.24 | 26.74 | 705 |
|  | Jul | 0.91 | 1.65 | 0.55 | 0.74 | 80.90 | 0.81 | 86.74 | 729 |
|  | Oct | 1.24 | 1.99 | 0.55 | 0.76 | 61.43 | 0.92 | 67.07 | 729 |
|  | Ann | 1.38 | 1.78 | 0.65 | 0.41 | 29.69 | 0.70 | 48.57 | 2876 |
| SO_2_ | Jan | 54.86 | 47.58 | 0.48 | -7.28 | -13.26 | 15.50 | 28.02 | 713 |
|  | Apr | 18.83 | 24.47 | 0.54 | 5.64 | 29.96 | 8.67 | 42.72 | 705 |
|  | Jul | 6.21 | 37.57 | 0.28 | 31.36 | 505.25 | 33.70 | 517.63 | 729 |
|  | Oct | 13.91 | 52.09 | 0.44 | 38.18 | 274.36 | 42.09 | 272.87 | 729 |
|  | Ann | 23.31 | 40.52 | 0.35 | 17.20 | 73.79 | 25.29 | 103.99 | 2876 |
| NO_2_ | Jan | 68.40 | 62.28 | 0.50 | -6.12 | -8.95 | 19.43 | 28.01 | 713 |
|  | Apr | 42.39 | 30.73 | 0.50 | -11.66 | -27.51 | 14.41 | 31.07 | 705 |
|  | Jul | 30.98 | 43.97 | 0.44 | 12.98 | 41.90 | 16.47 | 50.75 | 729 |
|  | Oct | 55.66 | 50.75 | 0.60 | -4.90 | -8.81 | 14.94 | 24.25 | 729 |
|  | Ann | 49.31 | 46.98 | 0.54 | -2.33 | -4.72 | 15.91 | 30.31 | 2876 |
| PM_2.5_ | Jan | 125.59 | 147.79 | 0.56 | 22.20 | 17.67 | 69.39 | 54.44 | 713 |
|  | Apr | 55.72 | 47.32 | 0.69 | -8.40 | -15.07 | 17.31 | 28.89 | 705 |
|  | Jul | 61.96 | 91.59 | 0.53 | 29.63 | 47.81 | 38.02 | 59.61 | 729 |
|  | Oct | 81.98 | 124.10 | 0.69 | 42.11 | 51.37 | 54.47 | 63.13 | 729 |
|  | Ann | 81.28 | 102.91 | 0.63 | 21.63 | 26.61 | 43.76 | 51.57 | 2876 |
| PM_10_ | Jan | 130.67 | 149.36 | 0.59 | 18.69 | 14.30 | 71.16 | 51.92 | 713 |
|  | Apr | 85.95 | 52.67 | 0.60 | -33.28 | -38.72 | 39.93 | 42.56 | 705 |
|  | Jul | 71.14 | 92.65 | 0.58 | 21.50 | 30.22 | 36.72 | 45.01 | 729 |
|  | Oct | 104.31 | 138.98 | 0.69 | 34.67 | 33.24 | 49.90 | 44.93 | 729 |
|  | Ann | 97.94 | 108.65 | 0.62 | 10.71 | 10.94 | 48.07 | 45.24 | 2876 |

*The units of Obs, Mod, MB, and ME for CO are mg m^-3^, for other species are μg m^-3^.

**Table S2.** Reduction percentages (%) and amounts (in parentheses) (Units of CO are mg m^-3^, and those for other species are μg m^-3^) of air pollutants for current emission control policies (Cases 2, 3 and 4) in Beijing on the basis of the simulations with resolution of 12×12 km.

| Species |  | (Case2-Case1)/Case1 (%) | (Case3-Case1)/Case1 (%) | (Case4-Case1)/Case1(%) |
| --- | --- | --- | --- | --- |
| CO | Jan | -20.76 (-0.54) | -20.70 (-0.54) | -20.42 (-0.53) |
|  | Apr | -19.51 (-0.20) | -18.89 (-0.19) | -19.34 (-0.20) |
|  | Jul | -20.69 (-0.36) | -20.46 (-0.35) | -20.74 (-0.36) |
|  | Oct | -20.26 (-0.43) | -20.28 (-0.43) | -20.01 (-0.43) |
|  | Ann | -20.44 (-0.39) | -20.29 (-0.38) | -20.24 (-0.38) |
| SO_2_ | Jan | -6.00 (-2.94) | -6.02 (-2.95) | -2.68 (-1.31) |
|  | Apr | -5.91 (-1.58) | -5.92 (-1.58) | -2.35 (-0.63) |
|  | Jul | -8.80 (-3.50) | -7.78 (-3.09) | -3.92 (-1.56) |
|  | Oct | -5.12 (-2.93) | -5.12 (-2.93) | -2.05 (-1.18) |
|  | Ann | -6.31 (-2.75) | -6.08 (-2.66) | -2.69 (-1.17) |
| NO_2_ | Jan | -6.74 (-4.32) | -6.68 (-4.28) | -3.01 (-1.93) |
|  | Apr | -7.13 (-2.39) | -7.17 (-2.40) | -3.20 (-1.07) |
|  | Jul | -9.03 (-4.20) | -8.32 (-3.87) | -4.75 (-2.21) |
|  | Oct | -3.38 (-1.88) | -3.36 (-1.88) | -1.57 (-0.88) |
|  | Ann | -6.33 (-3.19) | -6.15 (-3.10) | -3.01 (-1.52) |
| PM_2.5_ | Jan | -5.12 (-7.78) | -5.03 (-7.65) | -2.01 (-3.06) |
|  | Apr | -4.83 (-2.49) | -4.88 (-2.52) | -1.79 (-0.92) |
|  | Jul | -6.63 (-6.42) | -6.73 (-6.51) | -3.17 (-3.07) |
|  | Oct | -6.01 (-7.97) | -6.03 (-7.99) | -2.29 (-3.03) |
|  | Ann | -5.70 (-6.22) | -5.71 (-6.23) | -2.33 (-2.54) |
| PM_10_ | Jan | -4.80 (-7.73) | -4.77 (-7.67) | -1.84 (-2.97) |
|  | Apr | -4.42 (-2.55) | -4.48 (-2.58) | -1.64 (-0.94) |
|  | Jul | -6.72 (-7.11) | -6.51 (-6.89) | -3.27 (-3.45) |
|  | Oct | -5.39 (-8.06) | -5.43 (-8.11) | -2.08 (-3.11) |
|  | Ann | -5.36 (-6.40) | -5.33 (-6.35) | -2.20 (-2.63) |

**Table S3.** The same as Table S2 but for the newly-designed emission control policies (Cases 5, 6 and 7) in January.

| Species | (Case5-Case1)/Case1*100  (%) | (Case6-Case1)/Case1*100  (%) | (Case7-Case1)/Case1*100  (%) |
| --- | --- | --- | --- |
| CO | -22.98 (-0.60) | -27.73 (-0.73) | -23.26 (-0.61) |
| SO_2_ | -9.21 (-4.51) | -14.82 (-7.26) | -13.66 (-6.68) |
| NO_2_ | -8.89 (-5.70) | -11.10 (-7.11) | -9.29 (-5.95) |
| PM_2.5_ | -9.05 (-13.76) | -12.12 (-18.43) | -10.17 (-15.46) |
| PM_10_ | -8.62 (-13.87) | -11.75 (-18.71) | -10.61 (-17.07) |

**Table S4.** Contribution of thermal power plants in the BTH region to air pollutants in Beijing obtained on the basis of the results from the Case 8 and Case 1.

| Species | CO (%) | SO_2_ (%) | NO_2_ (%) | PM_2.5_ (%) | PM_10_ (%) |
| --- | --- | --- | --- | --- | --- |
| Jan | -39.42 | -23.55 | -22.02 | -27.07 | -26.59 |
| Apr | -34.12 | -22.20 | -22.99 | -21.03 | -21.82 |
| Jul | -35.16 | -23.43 | -21.63 | -22.19 | -20.97 |
| Oct | -38.73 | -22.97 | -24.73 | -22.16 | -24.16 |
| Ann | -37.63 | -23.09 | -23.02 | -23.78 | -24.04 |

**Table S5.** Reduction percentages and amounts (in parentheses, μg m^-3^) of PM_2.5_ of current emission control policies (cases 2, 3 and 4) for the other 12 cities in the BTH region on the basis of the simulations with the grid resolution of 12 km.

| Month | City | R2 | R3 | R4 | City | R2 | R3 | R4 |
| --- | --- | --- | --- | --- | --- | --- | --- | --- |
| Jan | Tianjin | -4.06(-6.98) | -4.47(-7.68) | -2.10(-3.61) | Langfang | -4.94(-9.29) | -4.92(-9.25) | -2.18(-4.09) |
| Apr |  | -3.38(-1.80) | -3.47(-1.85) | -1.38(-0.74) |  | -3.56(-2.07) | -3.62(-2.10) | -1.33(-0.77) |
| Jul |  | -6.57(-5.62) | -5.75(-4.92) | -2.12(-1.82) |  | -6.11(-6.18) | -5.17(-5.23) | -3.56(-3.60) |
| Oct |  | -3.43(-3.54) | -3.20(-3.31) | -1.30(-1.34) |  | -4.20(-5.76) | -4.11(-5.63) | -1.58(-2.16) |
| Annual |  | -4.33(-4.49) | -4.28(-4.44) | -1.81(-1.88) |  | -4.81(-5.85) | -4.58(-5.57) | -2.19(-2.67) |
| Jan | Baoding | -4.71(-10.69) | -4.76(-10.80) | -1.83(-4.16) | Qinhuangdao | -6.92(-4.89) | -6.91(-4.88) | -2.31(-1.63) |
| Apr |  | -4.38(-2.91) | -4.53(-3.02) | -1.45(-0.97) |  | -4.95(-1.24) | -4.88(-1.22) | -1.91(-0.45) |
| Jul |  | -5.96(-7.07) | -6.34(-7.52) | -1.61(-1.91) |  | -8.24(-3.07) | -7.19(-2.68) | -2.66(-0.99) |
| Oct |  | -5.52(-8.87) | -5.44(-8.74) | -2.04(-3.28) |  | -7.57(-4.06) | -7.69(-4.13) | -3.00(-1.61) |
| Annual |  | -5.02(-6.94) | -5.13(-7.10) | -1.75(-2.43) |  | -7.12(-3.34) | -6.93(-3.25) | -2.53(-1.19) |
| Jan | Cangzhou | -3.95(-5.90) | -4.17(-6.22) | -1.63(-2.43) | Shijiazhuang | -4.75(-10.42) | -4.76(-10.43) | -1.72(-3.77) |
| Apr |  | -3.32(-1.60) | -3.66(-1.76) | -1.11(-0.53) |  | -5.09(-3.85) | -4.66(-3.53) | -1.76(-1.33) |
| Jul |  | -8.86(-7.37) | -7.47(-6.22) | -3.50(-2.91) |  | -6.78(-8.62) | -6.87(-8.74) | -2.69(-3.42) |
| Oct |  | -2.73(-2.50) | -2.73(-2.50) | -1.09(-1.00) |  | -5.87(-10.11) | -5.87(-10.11) | -2.06(-3.54) |
| Annual |  | -4.66(-4.34) | -4.48(-4.17) | -1.85(-1.72) |  | -5.56(-8.29) | -5.53(-8.25) | -2.03(-3.03) |
| Jan | Chengde | -7.23(-3.27) | -7.19(-3.25) | -2.98(-1.35) | Tangshan | -4.74(-8.41) | -4.81(-8.54) | -1.94(-3.44) |
| Apr |  | -6.69(-1.52) | -6.71(-1.53) | -2.40(-0.55) |  | -3.48(-1.82) | -3.26(-1.74) | -1.25(-0.67) |
| Jul |  | -10.51(-4.52) | -10.64(-4.57) | -4.20(-1.80) |  | -4.34(-3.90) | -5.59(-5.03) | -0.39(-0.35) |
| Oct |  | -7.98(-4.25) | -7.95(-4.24) | -3.19(-1.70) |  | -4.54(-5.63) | -4.64(-5.75) | -1.82(-2.26) |
| Annual |  | -8.26(-3.41) | -8.28(-3.42) | -3.29(-1.36) |  | -4.45(-4.97) | -4.74(-5.29) | -1.51(-1.69) |
| Jan | Handan | -4.66(-9.82) | -4.69(-9.88) | -1.64(-3.46) | Xingtai | -5.41(-11.05) | -5.27(-10.76) | -1.92(-3.93) |
| Apr |  | -4.88(-3.78) | -4.81(-3.72) | -1.73(-1.34) |  | -5.84(-4.09) | -5.51(-3.85) | -1.98(-1.39) |
| Jul |  | -8.18(-9.26) | --8.99(-10.18) | -2.36(-2.67) |  | -8.02(-8.65) | -9.99(-10.77) | -1.63(-1.76) |
| Oct |  | -5.72(-8.59) | -5.72(-8.59) | -2.04(-3.07) |  | -6.41(-9.75) | -6.43(-9.79) | -2.18(-3.32) |
| Annual |  | -5.71(-7.89) | -5.88(-8.13) | -1.91(-2.65) |  | -6.29(-8.42) | -6.59(-8.84) | -1.95(-2.61) |
| Jan | Hengshui | -4.56(-7.66) | -4.56(-7.68) | -1.79(-3.02) | Zhangjiakou | -9.15(-3.28) | -9.13(-3.28) | -2.60(-0.93) |
| Apr |  | -4.03(-2.36) | -4.28(-2.51) | -1.70(-1.00) |  | -8.25(-1.61) | -8.09(-1.58) | -2.87(-0.56) |
| Jul |  | -5.73(-5.42) | -6.47(-6.13) | -2.07(-1.96) |  | -13.46(-4.12) | -14.31(-4.38) | -4.15(-1.27) |
| Oct |  | -3.38(-3.82) | -3.46(-3.91) | -1.39(-1.57) |  | -9.86(-3.88) | -9.90(-3.90) | -3.17(-1.25) |
| Annual |  | -4.43(-4.82) | -4.65(-5.06) | -1.73(-1.89) |  | -10.29(-3.24) | -10.49(-3.30) | -3.20(-1.01) |

*R2, R3 and R4 represent (Case 2-Case 1)/Case 1, (Case 3-Case 1)/Case 1, and (Case 4-Case 1)/Case 1, respectively.

**Table S6.** Model performance (Grid resolution: 36×36 km).

| Species |  | Obs | Mod | R | MB | NMB  (%) | ME | NME | N  pairs |
| --- | --- | --- | --- | --- | --- | --- | --- | --- | --- |
| CO | Jan | 2.54 | 1.88 | 0.42 | -0.66 | -25.91 | 1.03 | 40.05 | 713 |
|  | Apr | 0.82 | 0.92 | 0.51 | 0.10 | 12.29 | 0.26 | 28.66 | 705 |
|  | Jul | 0.91 | 1.74 | 0.51 | 0.83 | 91.46 | 0.92 | 98.80 | 729 |
|  | Oct | 1.23 | 1.85 | 0.59 | 0.62 | 50.47 | 0.83 | 61.36 | 729 |
|  | Ann | 1.37 | 1.60 | 0.46 | 0.23 | 16.72 | 0.78 | 53.98 | 2876 |
| SO_2_ | Jan | 54.26 | 40.78 | 0.36 | -13.48 | -24.84 | 18.83 | 33.96 | 713 |
|  | Apr | 18.91 | 25.21 | 0.53 | 6.30 | 33.30 | 8.25 | 40.45 | 705 |
|  | Jul | 6.21 | 38.99 | 0.28 | 32.79 | 528.26 | 35.30 | 542.12 | 729 |
|  | Oct | 13.87 | 49.49 | 0.45 | 35.62 | 256.93 | 39.22 | 255.38 | 729 |
|  | Ann | 23.18 | 38.72 | 0.25 | 15.54 | 67.06 | 26.52 | 109.90 | 2876 |
| NO_2_ | Jan | 68.57 | 60.50 | 0.41 | -8.07 | -11.77 | 21.58 | 31.04 | 713 |
|  | Apr | 43.38 | 31.46 | 0.42 | -11.92 | -27.47 | 16.25 | 34.21 | 705 |
|  | Jul | 31.02 | 45.37 | 0.42 | 14.35 | 46.27 | 18.13 | 55.81 | 729 |
|  | Oct | 55.65 | 48.09 | 0.56 | -7.56 | -13.58 | 17.12 | 27.80 | 729 |
|  | Ann | 49.60 | 46.40 | 0.46 | -3.20 | -6.45 | 18.13 | 34.09 | 2876 |
| PM_2.5_ | Jan | 125.62 | 112.35 | 0.37 | -13.27 | -10.56 | 73.28 | 57.48 | 713 |
|  | Apr | 55.79 | 51.63 | 0.65 | -4.16 | -7.46 | 34.80 | 31.10 | 705 |
|  | Jul | 61.96 | 100.64 | 0.51 | 38.68 | 62.42 | 46.18 | 72.40 | 729 |
|  | Oct | 82.39 | 123.73 | 0.76 | 41.34 | 50.18 | 49.14 | 56.65 | 729 |
|  | Ann | 81.41 | 97.38 | 0.54 | 15.97 | 19.62 | 48.30 | 56.40 | 2876 |
| PM_10_ | Jan | 130.67 | 113.95 | 0.41 | -16.72 | -12.79 | 76.30 | 55.66 | 713 |
|  | Apr | 86.53 | 57.04 | 0.54 | -29.49 | -34.08 | 37.71 | 39.94 | 705 |
|  | Jul | 70.79 | 99.21 | 0.54 | 28.42 | 40.14 | 44.96 | 55.37 | 729 |
|  | Oct | 105.34 | 135.87 | 0.75 | 30.53 | 28.98 | 41.14 | 36.65 | 729 |
|  | Ann | 98.25 | 101.82 | 0.53 | 3.57 | 3.63 | 51.62 | 45.14 | 2876 |

*The units of Obs, Mod and MB for CO are mg m^-3^, for other species are μg m^-3^.

**Table S7.** Model performance (Grid resolution: 4×4 km).

| Species |  | Obs | Mod | R | MB | NMB  (%) | ME | NME  (%) | N  pairs |
| --- | --- | --- | --- | --- | --- | --- | --- | --- | --- |
| CO | Jan | 2.54 | 2.68 | 0.77 | 0.14 | 5.39 | 0.72 | 27.82 | 713 |
|  | Apr | 0.82 | 0.90 | 0.60 | 0.08 | 9.97 | 0.24 | 26.73 | 705 |
|  | Jul | 0.91 | 1.54 | 0.50 | 0.63 | 69.07 | 0.71 | 76.30 | 729 |
|  | Oct | 1.24 | 1.85 | 0.55 | 0.61 | 49.27 | 0.87 | 63.12 | 729 |
|  | Ann | 1.38 | 1.74 | 0.73 | 0.37 | 26.75 | 0.63 | 43.93 | 2876 |
| SO_2_ | Jan | 54.71 | 52.34 | 0.52 | -2.37 | -4.33 | 14.86 | 26.58 | 713 |
|  | Apr | 18.83 | 24.50 | 0.47 | 5.67 | 30.13 | 9.77 | 48.12 | 705 |
|  | Jul | 6.21 | 36.91 | 0.26 | 30.70 | 494.71 | 32.99 | 506.70 | 729 |
|  | Oct | 13.86 | 50.11 | 0.32 | 36.25 | 261.46 | 39.70 | 258.57 | 729 |
|  | Ann | 23.27 | 41.04 | 0.39 | 17.77 | 76.39 | 24.67 | 101.65 | 2876 |
| NO_2_ | Jan | 68.38 | 62.98 | 0.57 | -5.40 | -7.90 | 16.93 | 24.42 | 713 |
|  | Apr | 42.39 | 30.57 | 0.54 | -11.82 | -27.89 | 14.29 | 30.82 | 705 |
|  | Jul | 30.98 | 43.07 | 0.45 | 12.08 | 39.00 | 15.33 | 47.24 | 729 |
|  | Oct | 55.66 | 51.46 | 0.60 | -4.20 | -7.54 | 13.94 | 22.64 | 729 |
|  | Ann | 49.30 | 47.07 | 0.58 | -2.24 | -4.54 | 15.08 | 28.75 | 2876 |
| PM_2.5_ | Jan | 125.55 | 157.68 | 0.64 | 32.13 | 25.59 | 63.29 | 49.67 | 713 |
|  | Apr | 55.72 | 44.41 | 0.65 | -11.31 | -20.30 | 19.04 | 31.77 | 705 |
|  | Jul | 61.96 | 80.59 | 0.41 | 18.62 | 30.05 | 34.47 | 54.04 | 729 |
|  | Oct | 81.98 | 110.72 | 0.54 | 28.74 | 35.06 | 53.98 | 62.56 | 729 |
|  | Ann | 81.27 | 98.47 | 0.63 | 17.20 | 21.16 | 42.58 | 50.12 | 2876 |
| PM_10_ | Jan | 130.54 | 162.94 | 0.66 | 32.40 | 24.82 | 67.18 | 49.06 | 713 |
|  | Apr | 85.95 | 49.58 | 0.59 | -36.37 | -42.32 | 41.50 | 44.23 | 705 |
|  | Jul | 71.14 | 85.17 | 0.51 | 14.02 | 19.71 | 33.50 | 41.06 | 729 |
|  | Oct | 104.07 | 125.04 | 0.55 | 20.97 | 20.14 | 50.57 | 45.64 | 729 |
|  | Ann | 97.85 | 105.83 | 0.62 | 7.99 | 8.16 | 47.81 | 45.03 | 2876 |

*The units of Obs, Mod and MB for CO are mg m^-3^, for other species are μg m^-3^.

**Table S8.** Reduction percentages (%) and amounts (in parentheses) (Units of CO are mg m^-3^, and those for other species are μg m^-3^) of air pollutants for current emission control policies (Cases 2, 3 and 4) in Beijing on the basis of the simulations with resolution of 36×36 km.

| Species |  | R2 | R3 | R4 |
| --- | --- | --- | --- | --- |
| CO | Jan | -19.78 (-0.38) | -20.03 (-1.72) | -19.74 (-0.38) |
|  | Apr | -17.78 (-0.18) | -17.26 (-0.18) | -17.74 (-0.18) |
|  | Jul | -21.46 (-0.39) | -21.02 (-0.38) | -19.77 (-0.36) |
|  | Oct | -20.15 (-0.41) | -20.07 (-0.41) | -19.85 (-0.40) |
|  | Ann | -20.09 (-0.35) | -19.92 (-0.35) | -19.55 (-0.34) |
| SO_2_ | Jan | -3.83 (-1.61) | -4.09 (-1.72) | -1.76 (-0.74) |
|  | Apr | -4.37 (-1.20) | -3.77 (-1.03) | -1.43 (-0.39) |
|  | Jul | -10.28(-4.24) | -9.22 (-3.80) | -5.00 (-2.06) |
|  | Oct | -4.51 (-2.45) | -4.48 (-2.44) | -1.67 (-0.91) |
|  | Ann | -5.68 (-2.41) | -5.37 (-2.28) | -2.44 (-1.04) |
| NO_2_ | Jan | -5.56 (-3.46) | -5.69 (-3.54) | -2.69 (-1.68) |
|  | Apr | -5.04 (-1.73) | -4.27 (-1.46) | -1.87 (-0.64) |
|  | Jul | -8.97 (-4.31) | -7.64 (-3.67) | -4.47 (-2.14) |
|  | Oct | -2.68 (-1.42) | -2.62 (-1.38) | -1.17 (-0.62) |
|  | Ann | -5.47 (-2.72) | -5.04 (-2.50) | -2.58 (-1.28) |
| PM_2.5_ | Jan | -4.47 (-5.16) | -4.66 (-5.38) | -1.79 (-2.07) |
|  | Apr | -3.92 (-2.21) | -3.41 (-1.92) | -1.21 (-0.68) |
|  | Jul | -8.72 (-9.28) | -8.39 (-8.98) | -3.25 (-3.45) |
|  | Oct | -5.40 (-7.13) | -5.29 (-6.99) | -2.04 (-2.69) |
|  | Ann | -5.88 (-6.21) | -5.70 (-6.01) | -2.21 (-2.33) |
| PM_10_ | Jan | -4.24 (-5.20) | -4.48 (-5.50) | -1.67 (-2.05) |
|  | Apr | -3.54 (-2.21) | -3.08 (-1.92) | -1.06 (-0.66) |
|  | Jul | -7.89 (-8.93) | -7.32 (-8.29) | -2.89 (-3.27) |
|  | Oct | -5.09 (-7.43) | -4.98 (-7.28) | -1.94 (-2.84) |
|  | Ann | -5.51 (-6.35) | -5.27 (-6.07) | -2.06 (-2.37) |

*R2, R3 and R4 represent (Case 2-Case 1)/Case 1, (Case 3-Case 1)/Case 1, and (Case 4-Case 1)/Case 1, respectively.

**Table S9.** Reduction percentages (%) and amounts (in parentheses) (Units of CO are mg m^-3^, and those for other species are μg m^-3^) of air pollutants for current emission control policies (Cases 2, 3 and 4) in Beijing on the basis of the simulations with resolution of 4×4 km.

| Species |  | R2 | R3 | R4 |
| --- | --- | --- | --- | --- |
| CO | Jan | -21.14 (-0.58) | -20.97 (-0.58) | -20.67 (-0.57) |
|  | Apr | -19.56 (-0.20) | -18.99 (-0.19) | -19.25 (-0.19) |
|  | Jul | -20.85 (-0.34) | -20.35 (-0.33) | -21.00 (-0.34) |
|  | Oct | -20.28 (-0.41) | -20.36 (-0.41) | -20.10 (-0.41) |
|  | Ann | -20.65 (-0.39) | -20.42 (-0.38) | -20.41 (-0.38) |
| SO_2_ | Jan | -7.33 (-3.94) | -7.20 (-3.88) | -2.90 (-1.56) |
|  | Apr | -6.90 (-1.84) | -6.83 (-1.82) | -2.61 (-0.70) |
|  | Jul | -10.20 (-3.98) | -9.99 (-3.90) | -4.97 (-1.94) |
|  | Oct | -5.56 (-3.06) | -5.66 (-3.12) | -2.26 (-1.25) |
|  | Ann | -7.37 (-3.23) | -7.30 (-3.20) | -3.13 (-1.37) |
| NO_2_ | Jan | -8.23 (-5.33) | -7.91 (-5.13) | -3.21 (-2.08) |
|  | Apr | -8.27 (-2.75) | -8.22 (-2.74) | -3.46 (-1.15) |
|  | Jul | -11.36 (-5.18) | -10.22 (-4.66) | -5.69 (-2.59) |
|  | Oct | -4.07 (-2.30) | -4.21 (-2.38) | -1.99 (-1.12) |
|  | Ann | -7.81 (-3.93) | -7.47 (-3.76) | -3.49 (-1.75) |
| PM_2.5_ | Jan | -5.66 (-9.18) | -5.51 (-8.94) | -2.13 (-3.46) |
|  | Apr | -5.16 (-2.50) | -5.12 (-2.48) | -1.70 (-0.82) |
|  | Jul | -8.04 (-6.84) | -7.66 (-6.52) | -4.30 (-3.66) |
|  | Oct | -6.41 (-7.58) | -6.44 (-7.61) | -2.49 (-2.94) |
|  | Ann | -6.31 (-6.59) | -6.18 (-6.45) | -2.63 (-2.75) |
| PM_10_ | Jan | -5.23 (-9.18) | -5.08 (-8.91) | -1.95 (-3.41) |
|  | Apr | -4.74 (-0.19) | -4.73 (-2.57) | -1.59 (-0.87) |
|  | Jul | -8.39 (-8.15) | -7.98 (-7.75) | -4.94 (-4.80) |
|  | Oct | -5.66 (-7.60) | -5.72 (-7.69) | -2.25 (-3.03) |
|  | Ann | -5.94 (-6.90) | -5.82 (-6.76) | -2.61 (-3.03) |

*R2, R3 and R4 represent (Case 2-Case 1)/Case 1, (Case 3-Case 1)/Case 1, and (Case 4-Case 1)/Case 1, respectively.

**Table S10.** Reduction percentages (%) and amounts (in parentheses) (Units of CO are mg m^-3^, and those for other species are μg m^-3^) of air pollutants for current emission control policies (Cases 2, 3 and 4) in Tianjin on the basis of the simulations with resolution of 12×12 km.

| Species |  | R2 | R3 | R4 |
| --- | --- | --- | --- | --- |
| CO | Jan | -21.27 (-0.73) | -21.68 (-0.74) | -21.42 (-0.73) |
|  | Apr | -19.89 (-0.24) | -19.47 (-0.23) | -19.86 (-0.24) |
|  | Jul | -20.90 (-0.36) | -20.31 (-0.35) | -20.39 (-0.36) |
|  | Oct | -20.58 (-0.40) | -20.37 (-0.40) | -20.24 (-0.40) |
|  | Ann | -20.83 (-0.44) | -20.77 (-0.43) | -20.70 (-0.43) |
| SO_2_ | Jan | -13.15 (-6.21) | -13.44 (-6.35) | -6.30 (-2.97) |
|  | Apr | -10.24 (-2.63) | -10.31 (-2.65) | -4.71 (-1.21) |
|  | Jul | -16.32 (-5.67) | -15.08 (-5.23) | -7.09 (-2.46) |
|  | Oct | -8.78 (-3.98) | -8.53 (-3.87) | -4.16 (-1.89) |
|  | Ann | -12.06 (-4.63 ) | -11.80 (-4.53) | -5.56 (-2.14) |
| NO_2_ | Jan | -14.60 (-8.23) | -15.23 (-8.59) | -7.78 (-4.39) |
|  | Apr | -13.38 (-3.49) | -13.38 (-3.49) | -6.33 (-1.65) |
|  | Jul | -18.05 (-5.97) | -17.51 (-5.79) | -9.12 (-3.02) |
|  | Oct | -11.00 (-4.35) | -10.73 (-4.24) | -5.60 (-2.21) |
|  | Ann | -14.19 (-5.51) | -14.23 (-5.53) | -7.25 (-2.82) |
| PM_10_ | Jan | -3.82 (-7.31) | -4.27 (-8.16) | -2.03 (-3.88) |
|  | Apr | -3.13 (-1.94) | -3.23 (-2.00) | -1.31 (-0.81) |
|  | Jul | -6.20 (-6.11) | -5.48 (-5.40) | -2.11 (-2.08) |
|  | Oct | -3.23 (-3.78) | -3.03 (-3.55) | -1.24 (-1.45) |
|  | Ann | -4.08 (-4.79) | -4.07 (-4.78) | -1.75 (-2.06) |

**-**

*R2, R3 and R4 represent (Case 2-Case 1)/Case 1, (Case 3-Case 1)/Case 1, and (Case 4-Case 1)/Case 1, respectively.

**Table S11.** Reduction percentages (%) and amounts (in parentheses) (Units of CO are mg m^-3^, and those for other species are μg m^-3^) of air pollutants for current emission control policies (Cases 2, 3 and 4) in Baoding on the basis of the simulations with resolution of 12×12 km.

| Species |  | R2 | R3 | R4 |
| --- | --- | --- | --- | --- |
| CO | Jan | -20.48 (-0.76) | -20.53 (-0.77) | -20.21 (-0.75) |
|  | Apr | -19.43 (-0.24) | -18.81 (-0.23) | -19.28 (-0.24) |
|  | Jul | -19.96 (-0.41) | -19.55 (-0.41) | -19.17 (-0.40) |
|  | Oct | -20.06 (-0.51) | -19.99 (-0.51) | -19.79 (-0.50) |
|  | Ann | -20.20 (-0.48) | -20.04 (-0.47) | -19.86 (-0.47) |
| SO_2_ | Jan | -7.04 (-4.02) | -7.03 (-4.01) | -2.64 (-1.51) |
|  | Apr | -6.98 (-2.00) | -6.95 (-1.99) | -2.61 (-0.75) |
|  | Jul | -9.48 (-3.60) | -9.22 (-3.50) | -1.74 (-0.66) |
|  | Oct | -6.23 (-3.64) | -6.15 (-3.59) | -2.28 (-1.33) |
|  | Ann | -7.64 (-3.32) | -7.54 (-3.28) | -2.47 (-1.07) |
| NO_2_ | Jan | -8.05 (-3.18) | -8.07 (-3.19) | -3.62 (-1.43) |
|  | Apr | -10.02 (-1.97) | -10.19 (-2.01) | -4.20 (-0.83) |
|  | Jul | -10.73 (-2.94) | -10.97 (-3.01) | -5.03 (-1.38) |
|  | Oct | -5.29 (-1.66) | -5.25 (-1.64) | -2.15 (-0.67) |
|  | Ann | -8.62 (-2.52) | -8.71 (-2.55) | -3.81 (-1.12) |
| PM_10_ | Jan | -4.36 (-10.88) | -4.42 (-11.03) | -1.70 (-4.25) |
|  | Apr | -3.98 (-2.96) | -4.15 (--3.09) | -1.32 (-0.98) |
|  | Jul | -5.47 (-7.27) | -5.82 (-7.74) | -1.42 (-1.89) |
|  | Oct | -5.06 (-9.04) | -4.99 (-8.91) | -1.88 (-3.36) |
|  | Ann | -4.61 (-7.08) | -4.73 (-7.26) | -1.60 (-2.46) |

*R2, R3 and R4 represent (Case 2-Case 1)/Case 1, (Case 3-Case 1)/Case 1, and (Case 4-Case 1)/Case 1, respectively.

**Table S12.** Reduction percentages (%) and amounts (in parentheses) (Units of CO are mg m^-3^, and those for other species are μg m^-3^) of air pollutants for current emission control policies (Cases 2, 3 and 4) in Cangzhou on the basis of the simulations with resolution of 12×12 km.

| Species |  | R2 | R3 | R4 |
| --- | --- | --- | --- | --- |
| CO | Jan | -20.39 (-0.48) | -20.42 (-0.48) | -20.19 (-0.47) |
|  | Apr | -17.92 (-0.14) | -17.55 (-0.13) | -17.95 (-0.14) |
|  | Jul | -20.63 (-0.25) | -19.52 (-0.22) | -19.61 (-0.23) |
|  | Oct | -18.95 (-0.24) | -18.92 (-0.24) | -18.82 (-0.24) |
|  | Ann | -19.77 (-0.27) | -19.49 (-0.27) | -19.44 (-0.27) |
| SO_2_ | Jan | -9.37 (-2.20) | -9.59 (-2.25) | -4.04 (-0.95) |
|  | Apr | -5.73 (-1.07) | -5.87 (-1.10) | -2.30 (-0.43) |
|  | Jul | -11.77 (-2.74) | -11.62 (-2.71) | -4.46 (-1.04) |
|  | Oct | -4.70 (-1.59) | -4.70 (-1.59) | -1.95 (-0.66) |
|  | Ann | -7.63 (-1.90) | -7.68 (-1.91) | -3.09 (-0.77) |
| NO_2_ | Jan | -13.16 (-3.87) | -13.23 (-3.89) | -6.06 (-1.78) |
|  | Apr | -8.44 (-1.14) | -8.61 (-1.16) | -3.64 (-0.49) |
|  | Jul | -8.47 (-1.37) | -8.11 (-1.32) | -3.35 (-0.54) |
|  | Oct | -7.23 (-1.60) | -7.17 (-1.59) | -3.13 (-0.69) |
|  | Ann | -9.79 (-2.00) | -9.76 (-1.99) | -4.31 (-0.88) |
| PM_10_ | Jan | -3.74 (-5.99) | -3.97 (-6.35) | -1.55 (-2.48) |
|  | Apr | -3.06 (-1.62) | -3.41 (-1.80) | -1.03 (-0.54) |
|  | Jul | -8.44 (-7.60) | -7.11 (-6.40) | -3.33 (-3.00) |
|  | Oct | -2.58 (-2.57) | -2.59 (-2.58) | -1.03 (-1.03) |
|  | Ann | -4.41 (-4.45) | -4.25 (-4.28) | -1.75 (-1.77) |

*R2, R3 and R4 represent (Case 2-Case 1)/Case 1, (Case 3-Case 1)/Case 1, and (Case 4-Case 1)/Case 1, respectively.

**Table S13.** The same as Table S12 but for Chengde.

| Species |  | R2 | R3 | R4 |
| --- | --- | --- | --- | --- |
| CO | Jan | -19.45 (-0.13) | -19.42 (-0.13) | -19.30 (-0.13) |
|  | Apr | -17.92 (-0.07) | -16.89 (-0.07) | -17.67 (-0.07) |
|  | Jul | -21.03 (-0.15) | -20.72 (-0.15) | -19.95 (-0.14) |
|  | Oct | -20.35 (-0.15) | -20.34 (-0.15) | -20.11 (-0.15) |
|  | Ann | -19.93 (-0.13) | -19.67 (-0.12) | -19.48 (-0.12) |
| SO_2_ | Jan | -11.55 (-1.50) | -11.56 (-1.50) | -4.60 (-0.60) |
|  | Apr | -9.90 (-0.86) | -9.93 (-0.86) | -3.68 (-0.32) |
|  | Jul | -17.82 (-2.28) | -18.24 (-2.33) | -7.11 (-0.91) |
|  | Oct | -9.60 (-1.89) | -9.59 (-1.88) | -3.73 (-0.73) |
|  | Ann | -12.05 (-1.64) | -12.15 (-1.65) | -4.73 (-0.64) |
| NO_2_ | Jan | -7.50 (-0.59) | -7.51 (-0.59) | -3.42 (-0.27) |
|  | Apr | -11.43 (-0.65) | -11.40 (-0.65) | -4.53 (-0.26) |
|  | Jul | -12.68 (-1.00) | -12.96 (-1.03) | -5.01 (-0.40) |
|  | Oct | -8.08 (-0.65) | -8.09 (-0.65) | -3.60 (-0.29) |
|  | Ann | -9.79 (-0.72) | -9.86 (-0.73) | -4.11 (-0.30) |
| PM_10_ | Jan | -6.81 (-3.29) | -6.79 (-3.28) | -2.81 (-1.36) |
|  | Apr | -6.37 (-1.54) | -6.41 (-1.55) | -2.29 (-0.55) |
|  | Jul | -10.02 (-4.65) | -10.18 (-4.72) | -3.97 (-1.84) |
|  | Oct | -7.61 (-4.31) | -7.60 (-4.30) | -3.05 (-1.73) |
|  | Ann | -7.86 (-3.47) | -7.90 (-3.49) | -3.13 (-1.38) |

*R2, R3 and R4 represent (Case 2-Case 1)/Case 1, (Case 3-Case 1)/Case 1, and (Case 4-Case 1)/Case 1, respectively.

**Table S14.** The same as Table S12 but for Handan.

| Species |  | R2 | R3 | R4 |
| --- | --- | --- | --- | --- |
| CO | Jan | -21.12 (-0.72) | -21.14 (-0.72) | -20.92 (-0.71) |
|  | Apr | -20.34 (-0.27) | -19.59 (-0.26) | -20.17 (-0.27) |
|  | Jul | -20.91 (-0.43) | -21.23 (-0.44) | -20.61 (-0.43) |
|  | Oct | -20.73 (-0.47) | -20.70 (-0.47) | -20.44 (-0.47) |
|  | Ann | -20.86 (-0.47) | -20.82 (-0.47) | -20.62 (-0.47) |
| SO_2_ | Jan | -10.54 (-5.88) | -10.58 (-5.90) | -3.64 (-2.03) |
|  | Apr | -8.03 (-2.95) | -8.09 (-2.97) | -2.81 (-1.03) |
|  | Jul | -14.96 (-6.76) | -14.03 (-6.34) | -4.89 (-2.21) |
|  | Oct | -7.66 (-4.85) | -7.64 (-4.83) | -2.65 (-1.68) |
|  | Ann | -10.16 (-5.12) | -9.96 (-5.02) | -3.45 (-1.74) |
| NO_2_ | Jan | -17.82 (-6.60) | -17.86 (-6.62) | -7.25 (-2.69) |
|  | Apr | -11.52 (-2.34) | -11.62 (-2.36) | -4.16 (-0.84) |
|  | Jul | -18.32 (-5.29) | -18.03 (-5.20) | -7.71 (-2.22) |
|  | Oct | -9.81 (-2.86) | -9.74 (-2.84) | -3.55 (-1.04) |
|  | Ann | -14.78 (-4.27) | -14.72 (-4.25) | -5.87 (-1.70) |
| PM_10_ | Jan | -4.36 (-10.01) | -4.42 (-4.42) | -1.54 (-3.53) |
|  | Apr | -4.57 (-3.89) | -4.54 (-4.54) | -1.64 (-1.39) |
|  | Jul | -7.60 (-9.63) | -8.45 (-8.45) | -2.22 (-2.82) |
|  | Oct | -5.32 (-8.81) | -5.34 (-5.34) | -1.91 (-3.16) |
|  | Ann | -5.33 (-8.12) | -5.54 (-5.54) | -1.80 (-2.74) |

*R2, R3 and R4 represent (Case 2-Case 1)/Case 1, (Case 3-Case 1)/Case 1, and (Case 4-Case 1)/Case 1, respectively.

**Table S15.** The same as Table S12 but for Hengshui.

| Species |  | R2 | R3 | R4 |
| --- | --- | --- | --- | --- |
| CO | Jan | -20.36 (-0.48) | -20.39 (-0.48) | -20.20 (-0.48) |
|  | Apr | -18.10 (-0.15) | -17.66 (-0.15) | -18.25 (-0.15) |
|  | Jul | -19.09 (-0.25) | -19.76 (-0.26) | -19.19 (-0.25) |
|  | Oct | -18.58 (-0.27) | -18.62 (-0.27) | -18.41 (-0.27) |
|  | Ann | -19.33 (-0.29) | -19.44 (-0.29) | -19.26 (-0.29) |
| SO_2_ | Jan | -10.81 (-2.70) | -10.89 (-2.72) | -4.00 (-1.00) |
|  | Apr | -6.67 (-1.41) | -6.96 (-1.47) | -2.43 (-0.51) |
|  | Jul | -12.31 (-2.94) | -12.80 (-3.06) | -4.69 (-1.12) |
|  | Oct | -5.45 (-1.90) | -5.53 (-1.93) | -2.08 (-0.73) |
|  | Ann | -8.51 (-2.24) | -8.72 (-2.30) | -3.19 (-0.84) |
| NO_2_ | Jan | -13.89 (-3.99) | -13.95 (-4.01) | -6.42 (-1.84) |
|  | Apr | -7.86 (-1.08) | -7.95 (-1.09) | -3.34 (-0.46) |
|  | Jul | -11.31 (-2.16) | -11.60 (-2.22) | -6.02 (-1.15) |
|  | Oct | -5.21 (-1.22) | -5.43 (-1.27) | -2.25 (-0.53) |
|  | Ann | -9.90 (-2.11) | -10.06 (-2.14) | -4.66 (-0.99) |
| PM_10_ | Jan | -4.37 (-7.78) | -4.39 (-7.82) | -1.73 (-3.08) |
|  | Apr | -3.82 (-2.40) | -4.10 (-2.57) | -1.63 (-1.02) |
|  | Jul | -5.44 (-5.52) | -6.26 (-6.36) | -2.00 (-2.03) |
|  | Oct | -3.21 (-3.89) | -3.29 (-4.00) | -1.32 (-1.60) |
|  | Ann | -4.22 (-4.90) | -4.47 (-5.19) | -1.66 (-1.93) |

*R2, R3 and R4 represent (Case 2-Case 1)/Case 1, (Case 3-Case 1)/Case 1, and (Case 4-Case 1)/Case 1, respectively.

**Table S16.** The same as Table S12 but for Langfang.

| Species |  | R2 | R3 | R4 |
| --- | --- | --- | --- | --- |
| CO | Jan | -20.45 (-0.68) | -20.44 (-0.68) | -20.26 (-0.67) |
|  | Apr | -18.88 (-0.22) | -18.42 (-0.21) | -18.91 (-0.22) |
|  | Jul | -19.17 (-0.35) | -18.36 (-0.33) | -19.55 (-0.35) |
|  | Oct | -19.77 (-0.45) | -19.66 (-0.44) | -19.39 (-0.44) |
|  | Ann | -19.79 (-0.42) | -19.53 (-0.42) | -19.69 (-0.42) |
| SO_2_ | Jan | -9.02 (-3.36) | -8.87 (-3.30) | -4.19 (-1.56) |
|  | Apr | -6.84 (-1.59) | -6.86 (-1.60) | -2.75 (-0.64) |
|  | Jul | -11.41 (-3.49) | -9.53 (-2.92) | -4.19 (-1.28) |
|  | Oct | -4.99 (-2.28) | -4.91 (-2.25) | -2.06 (-0.94) |
|  | Ann | -7.81 (-2.68) | -7.33 (-2.52) | -3.22 (-1.11) |
| NO_2_ | Jan | -6.86 (-3.59) | -6.79 (-3.55) | -3.88 (-2.03) |
|  | Apr | -8.00 (-2.19) | -8.05 (-2.21) | -3.75 (-1.03) |
|  | Jul | -8.81 (-3.04) | -9.55 (-3.30) | -5.08 (-1.75) |
|  | Oct | -3.83 (-1.73) | -3.65 (-1.64) | -1.75 (-0.79) |
|  | Ann | -6.59 (-2.63) | -6.68 (-2.67) | -3.49 (-1.39) |
| PM_10_ | Jan | -4.64 (-9.43) | -4.63 (-9.40) | -2.05 (-4.16) |
|  | Apr | -3.32 (-2.12) | -3.39 (-2.17) | -1.25 (-0.80) |
|  | Jul | -5.65 (-6.32) | -4.79 (-5.36) | -3.32 (-3.71) |
|  | Oct | -3.94 (-5.90) | -3.85 (-5.76) | -1.47 (-2.20) |
|  | Ann | -4.50 (-5.96) | -4.29 (-5.69) | -2.06 (-2.73) |

*R2, R3 and R4 represent (Case 2-Case 1)/Case 1, (Case 3-Case 1)/Case 1, and (Case 4-Case 1)/Case 1, respectively.

**Table S17.** The same as Table S12 but for Qinhuangdao.

| Species |  | R2 | R3 | R4 |
| --- | --- | --- | --- | --- |
| CO | Jan | -21.63 (-0.25) | -21.61 (-0.25) | -21.44 (-0.24) |
|  | Apr | -17.20 (-0.08) | -16.84 (-0.08) | -17.08 (-0.08) |
|  | Jul | -19.24 (-0.13) | -18.33 (-0.13) | -18.80 (-0.13) |
|  | Oct | -20.54 (-0.16) | -20.65 (-0.16) | -20.43 (-0.16) |
|  | Ann | -20.15 (-0.16) | -19.92 (-0.15) | -19.94 (-0.15) |
| SO_2_ | Jan | -11.94 (-2.29) | -11.96 (-2.29) | -4.50 (-0.86) |
|  | Apr | -6.85 (-0.57) | -6.82 (-0.57) | -2.62 (-0.22) |
|  | Jul | -14.18 (-1.73) | -12.63 (-1.54) | -4.91 (-0.60) |
|  | Oct | -9.26 (-1.99) | -9.35 (-2.01) | -3.61 (-0.78) |
|  | Ann | -10.75 (-1.66) | -10.48 (-1.61) | -4.02 (-0.62) |
| NO_2_ | Jan | -20.95 (-3.18) | -20.96 (-3.18) | -8.88 (-1.35) |
|  | Apr | -7.84 (-0.56) | -7.88 (-0.57) | -3.22 (-0.23) |
|  | Jul | -14.34 (-1.38) | -14.57 (-1.40) | -6.36 (-0.61) |
|  | Oct | -13.48 (-1.39) | -13.68 (-1.41) | -5.33 (-0.55) |
|  | Ann | -15.40 (-1.63) | -15.51 (-1.64) | -6.48 (-0.69) |
| PM_10_ | Jan | -6.35 (-4.93) | -6.37 (-4.95) | -2.12 (-1.65) |
|  | Apr | -4.09 (-1.26) | -4.03 (-1.25) | -1.58 (-0.49) |
|  | Jul | -7.06 (-3.12) | -6.20 (-2.74) | -2.20 (-0.97) |
|  | Oct | -6.82 (-4.13) | -6.96 (-4.22) | -2.72 (-1.65) |
|  | Ann | -6.32 (-3.38) | -6.18 (-3.31) | -2.24 (-1.20) |

*R2, R3 and R4 represent (Case 2-Case 1)/Case 1, (Case 3-Case 1)/Case 1, and (Case 4-Case 1)/Case 1, respectively.

**Table S18.** The same as Table S12 but for Shijiazhuang.

| Species |  | R2 | R3 | R4 |
| --- | --- | --- | --- | --- |
| CO | Jan | -21.60 (-0.86) | -21.60 (-0.86) | -21.39 (-0.85) |
|  | Apr | -20.83 (-0.31) | -19.85 (-0.30) | -20.57 (-0.31) |
|  | Jul | -20.91 (-0.50) | -20.23 (-0.49) | -20.66 (-0.50) |
|  | Oct | -20.63 (-0.60) | -20.99 (-0.61) | -20.63 (-0.60) |
|  | Ann | -21.17 (-0.57) | -20.89 (-0.57) | -20.90 (-0.57) |
| SO_2_ | Jan | -10.49 (-6.96) | -10.47 (-6.94) | -3.75 (-2.49) |
|  | Apr | -8.64 (-3.31) | -8.65 (-3.32) | -3.08 (-1.18) |
|  | Jul | -13.08 (-6.69) | -12.06 (-6.17) | -4.31 (-2.21) |
|  | Oct | -7.05 (-5.11) | -7.10 (-5.15) | -2.44 (-1.77) |
|  | Ann | -9.64 (-5.53) | -9.42 (-5.40) | -3.34 (-1.91) |
| NO_2_ | Jan | -15.81 (-7.23) | -15.77 (-7.21) | -6.15 (-2.81) |
|  | Apr | -11.87 (-2.77) | -11.72 (-2.74) | -4.61 (-1.08) |
|  | Jul | -13.75 (-4.24) | -12.85 (-3.96) | -5.59 (-1.72) |
|  | Oct | -6.35 (-2.22) | -6.31 (-2.21) | -2.33 (-0.81) |
|  | Ann | -12.14 (-4.10) | -11.89 (-4.02) | -4.74 (-1.60) |
| PM_10_ | Jan | -4.42 (-10.65) | -4.45 (-10.72) | -1.61 (-3.87) |
|  | Apr | -4.67 (-3.96) | -4.30 (-3.65) | -1.62 (-1.37) |
|  | Jul | -6.26 (-8.97) | -6.32 (-9.06) | -2.50 (-3.58) |
|  | Oct | -5.39 (-10.35) | -5.40 (-10.38) | -1.89 (-3.62) |
|  | Ann | -5.14 (-8.53) | -5.12 (-5.12) | -1.89 (-3.13) |

*R2, R3 and R4 represent (Case 2-Case 1)/Case 1, (Case 3-Case 1)/Case 1, and (Case 4-Case 1)/Case 1, respectively.

**Table S19.** The same as Table S12 but for Tangshan.

| Species |  | R2 | R3 | R4 |
| --- | --- | --- | --- | --- |
| CO | Jan | -22.06 (-0.87) | -22.08 (-0.87) | -21.83 (-0.86) |
|  | Apr | -20.83 (-0.28) | -20.34 (-0.28) | -20.79 (-0.28) |
|  | Jul | -20.00 (-0.42) | -21.64 (-0.45) | -20.06 (-0.42) |
|  | Oct | -21.40 (-0.58) | -21.45 (-0.58) | -21.15 (-0.57) |
|  | Ann | -21.29 (-0.54) | -21.59 (-0.55) | -21.14 (-0.54) |
| SO_2_ | Jan | -9.82 (-5.21) | -9.78 (-5.19) | -3.92 (-2.08) |
|  | Apr | -7.84 (-2.00) | -7.77 (-1.98) | -2.93 (-0.75) |
|  | Jul | -12.04 (-4.32) | -13.03 (-4.68) | -3.71 (-1.33) |
|  | Oct | -7.05 (-3.59) | -6.91 (-3.52) | -2.72 (-1.39) |
|  | Ann | -9.13 (-3.79) | -9.27 (-3.85) | -3.34 (-1.39) |
| NO_2_ | Jan | -17.20 (-7.97) | -17.16 (-7.95) | -7.81 (-3.62) |
|  | Apr | -13.40 (-2.80) | -13.33 (-2.78) | -5.37 (-1.12) |
|  | Jul | -17.61 (-5.13) | -19.35 (-5.63) | -7.64 (-2.22) |
|  | Oct | -10.72 (-3.72) | -10.77 (-3.73) | -4.58 (-1.59) |
|  | Ann | -14.94 (-4.90) | -15.31 (-5.02) | -6.51 (-2.14) |
| PM_10_ | Jan | -4.29 (-8.71) | -4.37 (-8.89) | -1.77 (-3.59) |
|  | Apr | -3.11 (-1.97) | -2.93 (-1.85) | -1.13 (-0.72) |
|  | Jul | -3.76 (-3.97) | -5.31 (-5.61) | -0.26 (-0.28) |
|  | Oct | -4.06 (-5.85) | -4.18 (-6.03) | -1.64 (-2.36) |
|  | Ann | -3.97 (-5.15) | -4.34 (-5.62) | -1.35 (-1.74) |

*R2, R3 and R4 represent (Case 2-Case 1)/Case 1, (Case 3-Case 1)/Case 1, and (Case 4-Case 1)/Case 1, respectively.

**Table S20.** The same as Table S12 but for Xingtai.

| Species |  | R2 | R3 | R4 |
| --- | --- | --- | --- | --- |
| CO | Jan | -21.28 (-0.71) | -21.24 (-0.71) | -21.12 (-0.71) |
|  | Apr | -20.50 (-0.24) | -19.38 (-0.23) | -20.27 (-0.24) |
|  | Jul | -19.68 (-0.37) | -20.20 (-0.38) | -19.32 (-0.36) |
|  | Oct | -20.93 (-0.48) | -20.89 (-0.48) | -20.61 (-0.47) |
|  | Ann | -20.74 (-0.45) | -20.68 (-0.45) | -20.49 (-0.45) |
| SO_2_ | Jan | -11.36 (-6.05) | -11.29 (-6.02) | -4.04 (-2.15) |
|  | Apr | -9.87 (-3.28) | -9.70 (-3.22) | -3.51 (-1.16) |
|  | Jul | -13.89 (-5.67) | -13.58 (-5.54) | -3.98 (-1.62) |
|  | Oct | -8.12 (-5.05) | -8.10 (-5.05) | -2.81 (-1.75) |
|  | Ann | -10.55 (-5.02) | -10.43 (-4.97) | -3.52 (-1.68) |
| NO_2_ | Jan | -20.03 (-6.76) | -20.04 (-6.76) | -8.28 (-2.79) |
|  | Apr | -14.78 (-2.57) | -14.75 (-2.57) | -5.46 (-0.95) |
|  | Jul | -18.57 (-4.48) | -18.18 (-4.39) | -6.64 (-1.60) |
|  | Oct | -9.33 (-2.46) | -9.27 (-2.44) | -3.40 (-0.90) |
|  | Ann | -15.94 (-4.06) | -15.83 (-4.03) | -6.11 (-1.56) |
| PM_10_ | Jan | -5.07 (-11.24) | -4.96 (-11.00) | -1.81 (-4.02) |
|  | Apr | -5.52 (-4.20) | -5.22 (-3.97) | -1.88 (-1.44) |
|  | Jul | -7.43 (-8.86) | -9.43 (-11.24) | -1.49 (-1.78) |
|  | Oct | -5.98 (-9.96) | -6.02 (-10.04) | -2.03 (-3.40) |
|  | Ann | -5.87 (-8.61) | -6.22 (-9.11) | -1.82 (-2.67) |

*R2, R3 and R4 represent (Case 2-Case 1)/Case 1, (Case 3-Case 1)/Case 1, and (Case 4-Case 1)/Case 1, respectively.

**Table S21.** The same as Table S12 but for Zhangjiakou.

| Species |  | R2 | R3 | R4 |
| --- | --- | --- | --- | --- |
| CO | Jan | -18.71 (-0.11) | -18.70 (-0.11) | -18.51 (-0.11) |
|  | Apr | -18.02 (-0.07) | -16.94 (-0.06) | -17.79 (-0.07) |
|  | Jul | -20.40 (-0.11) | -20.10 (-0.11) | -19.24 (-0.10) |
|  | Oct | -19.57 (-0.11) | -19.59 (-0.11) | -19.31 (-0.11) |
|  | Ann | -19.27 (-0.10) | -19.01 (-0.10) | -18.80 (-0.10) |
| SO_2_ | Jan | -15.93 (-2.69) | -15.95 (-2.70) | -5.58 (-0.94) |
|  | Apr | -15.95 (-1.65) | -16.13 (-1.67) | -5.53 (-0.57) |
|  | Jul | -36.47 (-5.31) | -36.31 (-5.29) | -11.78 (-1.71) |
|  | Oct | -15.85 (-3.45) | -15.80 (-3.44) | -5.41 (-1.18) |
|  | Ann | -20.59 (-3.29) | -20.57 (-3.29) | -6.93 (-1.11) |
| NO_2_ | Jan | -28.33 (-3.08) | -28.33 (-3.08) | -10.96 (-1.19) |
|  | Apr | -30.81 (-2.19) | -30.77 (-2.19) | -11.00 (-0.78) |
|  | Jul | -52.48 (-6.88) | -51.38 (-6.73) | -18.28 (-2.39) |
|  | Oct | -35.51 (-3.65) | -35.49 (-3.65) | -12.84 (-1.32) |
|  | Ann | -38.24 (-3.96) | -37.88 (-3.93) | -13.77 (-1.43) |
| PM_10_ | Jan | -8.61 (-3.36) | -8.66 (-3.38) | -2.47 (-0.96) |
|  | Apr | -7.96 (-1.66) | -7.88 (-1.64) | -2.79 (-0.58) |
|  | Jul | -13.11 (-4.36) | -14.07 (-4.68) | -4.11 (-1.37) |
|  | Oct | -9.49 (-4.01) | -9.61 (-4.06) | -3.08 (-1.30) |
|  | Ann | -9.90 (-3.37) | -10.18 (-3.46) | -3.12 (-1.06) |

*R2, R3 and R4 represent (Case 2-Case 1)/Case 1, (Case 3-Case 1)/Case 1, and (Case 4-Case 1)/Case 1, respectively.
